# Quantitative Proteomics of the Root of Transgenic Wheat Expressing *TaBWPR-1.2* Genes in Response to Waterlogging

**DOI:** 10.3390/proteomes2040485

**Published:** 2014-11-04

**Authors:** Emdadul Haque, Fumitaka Abe, Masahiko Mori, Yohei Nanjo, Setsuko Komatsu, Atsushi Oyanagi, Kentaro Kawaguchi

**Affiliations:** NARO Institute of Crop Science (NICS), National Agriculture and Food Research Organization (NARO), 2-1-18 Kannondai, Tsukuba, Ibaraki 305-8518, Japan; E-Mails: haque@yokohama-cu.ac.jp (E.H.); mmmori@obihiro.ac.jp (M.M.); ynanjo@affrc.go.jp (Y.N.); skomatsu@affrc.go.jp (S.K.); oyanagi@affrc.go.jp (A.O.); kentaro@affrc.go.jp (K.K.)

**Keywords:** transgenic wheat, pathogenesis-related protein-1.2, proteomics, seminal roots, waterlogging

## Abstract

Once candidate genes are available, the application of genetic transformation plays a major part to study their function in plants for adaptation to respective environmental stresses, including waterlogging (WL). The introduction of stress-inducible genes into wheat remains difficult because of low transformation and plant regeneration efficiencies and expression variability and instability. Earlier, we found two cDNAs encoding WL stress-responsive wheat pathogenesis-related proteins 1.2 (*TaBWPR-1.2*), *TaBWPR-1.2#2* and *TaBWPR-1.2#*13. Using microprojectile bombardment, both cDNAs were introduced into “Bobwhite”. Despite low transformation efficiency, four independent T_2_ homozygous lines for each gene were isolated, where transgenes were ubiquitously and variously expressed. The highest transgene expression was obtained in *Ubi:TaBWPR-1.2#2* L#11a and *Ubi:TaBWPR-1.2#13* L#4a. Using quantitative proteomics, the root proteins of L#11a were analyzed to explore possible physiological pathways regulated by TaBWPR-1.2 under normal and waterlogged conditions. In L#11a, the abundance of proteasome subunit alpha type-3 decreased under normal conditions, whereas that of ferredoxin precursor and elongation factor-2 increased under waterlogged conditions in comparison with normal plants. Proteomic results suggest that L#11a is one of the engineered wheat plants where TaBWPR-1.2#2 is most probably involved in proteolysis, protein synthesis and alteration in the energy pathway in root tissues via the above proteins in order to gain metabolic adjustment to WL.

## 1. Introduction

In the last two decades, genetic transformation has become a powerful tool to transfer new genes into crop plants. This approach offers an attractive alternative to conventional breeding, because specific traits can be transferred into selected genotypes without adverse effects on desirable genetic backgrounds. Wheat (*Triticum aestivum* L.) is one of the most important crops that feeds the growing world population. Its production is predicted to decline (along with that of other cereals) due to adverse environments. Among cereals, wheat was the last to be genetically modified, because of inherent difficulties associated with gene delivery into regenerable explants and recovery of transformants; wheat, particularly hexaploid, has a larger genome than other cereals [1,2,3]. Transgenic wheat lines producing some proteins involved in development have been obtained [2,3,4,5], and corresponding genes, proteins or metabolites have been analyzed; yet, this approach is still a challenge for stress-inducible genes [6,7,8].

Transcription of stress-inducible genes depends on the strength and duration of stimuli. These genes can be divided into early and late responsive [9]. To the best of our knowledge, no well-characterized wheat-derived promoters for constitutive, tissue-specific or stress-inducible expression are available. In wheat, the maize ubiquitin promoter and intron (*Ubi*) [10] and the rice actin promoter with the 5' intron (*Act1*) [11] appear to result in the highest and most stable constitutive expression. Position effects, the developmental stage [12] and, rarely, stress [13] may affect *Ubi* activity in transgenic wheat lines. Recently, promising stress-inducible promoters, such as *Arabidopsis rd29A* [8], maize *Rab17* [6] and barley *HvDhn4s* [7], have been used to study the effect of drought, but these promoters may be not efficient in heterologous systems. To circumvent these problems, some wheat genes, particularly stress-inducible ones, have been overexpressed in other plants [14,15].

Proteomic techniques in conjunction with mass spectrometry (MS), including gel-based and gel-free proteomics, enable comparative quantitative protein profiling. Because of the disadvantages of gel-based proteomics (labor intensiveness, low sensitivity and reproducibility and the inability to characterize complete proteomes), gel-free proteomics has become a valuable tool for functional analyses of particular biological processes or responses to the environment [5,16,17].

Plants’ ability to tolerate water stresses, such as drought and waterlogging (WL), is crucial for agricultural production worldwide. Stress environments trigger a wide variety of plant responses through sensing, signaling and adaption. Soil WL has been a serious environmental stress that imposes on plant growth and productivity [18]. To design molecular mechanism for WL tolerance, elucidation of cellular systems involved in responses and adaptations have been required to efficiently discover key genes to be applied to engineer its tolerance. For this purpose, here, we focus on response and tolerance systems against WL in wheat plants. 

The hexaploid wheat genome contains 23 pathogenesis-related (PR) protein-1-like genes, designated as TaPR-1.1 to 20 [19]. Among them, the deduced TaPR-1.20 protein sequence was highly identical to that encoded by the *TaPR-1.2* gene [20]. TaPR-1.2 (TaPR-1.20) is not a marker for systemic acquired resistance [19], but a stress (aluminum, humidity)-responsive gene [19,21]. Although little is known about *PR-1.2* gene expression and protein production in root in response to environmental stresses, relevant information has been obtained for other PR families. For example, PR-10 proteins in rice and maize were found to respond to drought and cold, respectively [22,23,24]. In a previous study on morphological adaptation to WL in the seminal roots of hexaploid spring wheat “Bobwhite SH 98 26” [25], we found that levels of a TaPR-1.2 significantly increased during lysigenous aerenchyma formation [26]. We thought that there was a relationship between TaPR-1.2 and WL response and/or aerenchyma tissue formation in wheat seminal roots. Very recently, we identified two TaPR-1.2 cDNAs, *TaBWPR-1.2#2* (AB711115) and *TaBWPR-1.2#13* (AB711116), from the seminal root of Bobwhite as WL-responsive at the RNA and protein levels [27]. These clones differ by the presence or absence of two amino acids (FA) at positions 164–165 and one amino acid, lysine (“K”; *i.e.*, a positive charge), at the C-terminal end. However, the functional differences between these two TaBWPR-1.2 clones in wheat were unknown. Moreover, wheat plants transformed with WL stress-responsive gene(s) are not yet available.

In the present work, we used the biolistic approach to transform wheat cultivar “Bobwhite SH 98 26” and produced homozygous transgenic lines overexpressing *TaBWPR-1.2#2* or *TaBWPR-1.2#13* under the control of the *Ubi* promoter. To explore the physiological pathway of *TaBWPR-1.2*, we compared protein abundance in control and transgenic wheat seminal roots under control and WL conditions by gel-free proteomics. This work may be useful for those who attempt to produce transgenic wheat plants and for those interested in the role of PR-1.2 proteins in wheat.

## 2. Experimental Section

### 2.1. Construct Preparation

The original TaBWPR-1.2#2 and TaBWPR-1.2#13 cDNAs were cloned into the pCRII-TOPO vector (Invitrogen, Carlsbad, CA, USA) and were described previously [27]. The coding regions were then amplified with a primer set containing the BamHI sites, and the fragments were inserted into the BamHI site of the plasmid, pAHC17 [28]. The two constructs were designated as pUbi:TaBWPR-1.2#2 and pUbi:TaBWPR-1.2#13.

### 2.2. Plant Material Preparation for Transformation

Spring wheat (*Triticum aestivum* L. cv. Bobwhite 98 26) [29] was used in all experiments. Seeds were sown (four seeds per 18-cm pot) in a 2:1 mixture of Sakata Soil Mix (Sakata Seed Corp., Yokohama, Japan) and Kureha fertilized granulated soil (Kureha Corp., Tokyo, Japan). Plants were grown in a greenhouse at 17 °C (day)/10 °C (night) with an 8-h photoperiod for 12 weeks and then transferred into a controlled-environment chamber at 20/13 °C with a 16-h photoperiod (750 μmol·m^−2^·s) and 55%–65% relative humidity. Tillers were harvested 13–15 days after anthesis by cutting below the third node of the tiller with three leaves retained and kept at 5 °C without water supply for 5–7 days.

### 2.3. Isolation of Scutellar Tissues from Immature Embryos

Immature caryopses were collected from the spikelets 10–12 days after anthesis, rinsed with 70% ethanol, surface-sterilized in sodium hypochlorite solution (0.5% v/v available chlorine) containing 0.1% v/v Tween 20 for 15 min and then rinsed three times with sterile distilled water. Immature embryos were isolated aseptically under a dissecting microscope, and the entire axis of the embryos was removed by a fine blade. Isolated scutellar tissues were cultured, scutellum side up, on callus induction medium containing 0.2 M mannitol (CI-0.2Man) at 25 °C in the dark for 3–4 h before bombardment.

### 2.4. Biolistic Transformation

Scutellar tissues were bombarded with each plasmid. Plasmid DNA was prepared using a Qiagen Maxi Kit (Qiagen, Hilden, Germany). The plasmid pUba [30], carrying the *bar* gene that confers resistance to the herbicide, phosphinothricin, was co-bombarded with each plasmid, pUbi:TaBWPR-1.2#2 or pUbi:TaBWPR-1.2#13, in a 1:1.5 molar ratio. Plasmid DNA (total 5 μg) was precipitated onto gold particles (2 mg; 1.0 μm in diameter) in the presence of 1 M CaCl_2_ and 16 mM spermidine, and then, the DNA-gold particles were washed twice with ethanol and resuspended in 100 μL of ethanol. For each bombardment, 5 μL of the DNA-gold suspension was used (100 μg particles per shot). Particles were bombarded with a PDS 1000/He particle delivery system (Bio-Rad, Hercules, CA, USA). The target tissues were placed 5.5 cm from the stopping screen at a helium pressure of 6.2 MPa.

### 2.5. Tissue Culture and Selection of Transgenic Plants

The composition of tissue culture media is listed in Supplementary Table 1. At 2 days after bombardment, scutellar tissues (16 per 90-mm plate) were cultured on callus maintenance medium containing 3 mg·L^−1^ phosphinothricin (CM-3P) for 3 weeks. The explants were transferred to shoot growth medium containing 1 mg·L^−1^ phosphinothricin (SG-1P) (8 calluses per plate) under illumination for 3 weeks for shoot regeneration and then to root growth medium containing 3 mg·L^−1^ phosphinothricin (RG-3P) for a further 3 weeks for root regeneration. Plants resistant to phosphinothricin were transferred to soil.

### 2.6. PCR Analysis of Transgenic Plants

DNA-PCR and reverse-transcription PCR (RT-PCR) were used to screen transgenic plants. Genomic DNA was isolated from leaf tips as described by [31]. Total RNA was isolated from leaf tips as described by [27]. The forward primer for DNA-PCR was designed within the *Ubi* promoter (5'-ttagccctgccttcatacgc-3'). That for RT-PCR was designed between *Ubi*, and the sequence was identical in *TaBWPR-1.2#2* and *TaBWPR-1.2#13* region (5'-actctagaggatccccatgg-3'). The reverse primers for DNA-PCR and RT-PCR corresponded to the unique sequences of *TaBWPR-1.2#2* (5'-ttgttgtcccatgccacgg-3') and *TaBWPR-1.2#13* (5'-ctgttgtcccacgtcacag-3').

### 2.7. Analysis of Gene Expression in Different Organs by RT-PCR

Seeds germinated on wet filter paper in a glass Petri dish for 4 days were raised in either big glass Petri dishes (height 6 cm × diameter 9 cm; As One Stock, Tokyo, Japan) for another 4 days, then the leaf, the root base (1 cm), the middle part of the root (3–5 cm) and the root tip (1 cm) were collected; or 30 cm-long well-drained pots [25] in the phytotron chamber; the whole leaf and root were collected 15 days later, and spikes were collected 90 days later (before anthesis). Total RNA was extracted from wheat organs as described previously [27]. One-step PCR was performed with the PrimeScript RT reagent kit (Takara, Kyoto, Japan) in a 10-µL reaction volume (200 ng of total RNA). One-step RT-PCR was performed using a PCR System (Takara) under conditions of 50 °C for 30 m followed by 33 cycles of 95 °C for 30 s, 60 °C for 30 s and 72 °C for 30 s with gene-specific primers as above.

### 2.8. Gene Expression Analysis by qRT-PCR in Homozygous Transformants under WL

Homozygous transformants and their null-segregants were grown in pots in a phytotron chamber for 7 days, followed by 5 days of WL [26]. Whole-root samples were prepared from normal and waterlogged 12-day-old plants and stored at −80 °C. qRT-PCR was performed according to [32], as slightly modified by [27]. The primer design and amplification efficiency are also described in detail in these two publications [27,32]. To detect the transgenes, we used the gene-specific primers described above. To detect endogenous genes, we used the primers 5'-cttgacgccgaagcctagta-3' (forward) and 5'-gccggaatgtgtgcttattt-3' (reverse) for *TaBWPR-1.2#2* and 5'-cgcactggtcatagtcatgg-3' (forward) and 5'-ctgttgtcccacgtcacag-3' (reverse) for *TaBWPR-1.2#13*. An actin gene was used as an internal control. All RT- and qRT-PCR experiments were performed in biological triplicates and technical triplicates.

### 2.9. Protein Extraction and Immunoblot Analysis with Rice Anti-PR-1 Antibody

Homozygous lines and control (12-day-old plants) were subjected to 5-day WL, and whole roots were collected as samples. Samples were ground in SDS sample buffer consisting of 60 mM Tris-HCl (pH 6.8), 2% SDS, 10% glycerol and 5% 2-mercaptoethanol. After centrifugation, supernatant was separated on a 12% SDS polyacrylamide electrophoresis gel. Immunoblot analysis was performed according to [27] with an anti-rice PR-1 antibody [33].

### 2.10. Preparation of Proteins for Mass Spectrometry (MS)

Protein concentration in the extracts was estimated by a Pierce 660 nm Protein Assay Kit with the Ionic Detergent Compatibility Reagent (Thermo Fisher Scientific, Waltham, MA, USA). Detergent was removed from the extracted proteins (100 μg) by chloroform-methanol extraction as follows. Samples (adjusted to 100 μL) were mixed consecutively with methanol (400 μL), chloroform (100 μL) and water (300 μL) and centrifuged at 20,000× *g* for 5 min for phase separation. The upper (aqueous) phase was discarded, and methanol (300 μL) was added to the organic phase. The samples were centrifuged again at 20,000× *g* for 5 min; the supernatants were discarded and the pellets dried. Proteins were reduced with 50 mM dithiothreitol for 1 h at 56 °C, alkylated with 50 mM iodoacetamide for 1 h at 37 °C in the dark and digested with trypsin and lysyl endopeptidase at a 1:100 enzyme/protein ratio for 16 h at 37 °C. The resulting peptides were acidified with formic acid to pH < 3, desalted with a C18-pipette tip and analyzed by MS.

### 2.11. Data Acquisition by Nano-Liquid Chromatography (LC) MS/MS

Peptides were analyzed on a nanospray LTQ XL Orbitrap mass spectrometer (Thermo Fisher Scientific) operated in data-dependent acquisition mode with Xcalibur software (version 2.0.7, Thermo Fisher Scientific). Using an Ultimate 3000 nanoLC system (Dionex, Germering, Gemany), peptides in 0.1% formic acid were loaded onto a C18 PepMap trap column (300 µm ID × 5 mm, Dionex), eluted and separated on a C18 Tip column (75 µm ID × 120 mm nano-HPLC capillary column NTTC-360/75-3; Nikkyo Technos, Tokyo, Japan) in a linear acetonitrile gradient (8%–30% in 120 min) in 0.1% formic acid at a flow rate of 200 nL/min. A spray voltage of 1.5 kV was used. Full-scan mass spectra were acquired over a mass range of 400–1500 *m*/*z* with a resolution of 30,000. The lock mass function was used to obtain high mass accuracy. The ten most intense precursor ions were selected for collision-induced fragmentation in the linear ion trap at a normalized collision energy of 35%. Dynamic exclusion was used within 90 s to prevent repetitive selection of the same peptides.

### 2.12. Protein Identification

Proteins were identified by the Mascot search engine (version 2.3.0.2, Matrix Science, London, U.K.) through Mascot Daemon client software (version 2.3.2, Matrix Science) using a customized *T. aestivum* database containing 21,690 protein sequences. The protein sequences were obtained from the Triticeae Full-Length CDS database (6146 sequences) [34], NCBI database (10,690 sequences) [35] and UniProt database (4854 sequences) [36]. The parameters used in Mascot searches were as follows: cysteine carbamidomethylation was set as a fixed modification, and methionine oxidation was set as a variable modification. Trypsin was specified as the proteolytic enzyme, and one missed cleavage was allowed. Peptide mass tolerance was set at 5 ppm. Fragment mass tolerance was set at 0.5 Da, and peptide charge was set at +2, +3 or +4. An automatic decoy database search was performed as part of the search. Mascot results were filtered with Mascot Percolator to improve the accuracy and sensitivity of peptide identification. False discovery rates for peptide identification were <1.0% in all searches. The Mascot results were exported in XML format for SIEVE (version 2.0, Thermo Fisher Scientific) analysis.

### 2.13. Analysis of Differential Protein Abundance Using Acquired MS Data

Analysis of protein abundance was performed by using the label-free quantification package, SIEVE (Thermo Fisher Scientific), to compare the relative abundance of peptides and proteins in the control and experimental groups, as previously described by [37]. It is important to note that we performed this study in a phytotron chamber where the stress level is mild (low light intensity and low temperature compared to a greenhouse). Therefore, the thresholds for fold changes in protein quantities in transgenic *vs.* non-transgenic samples were set at >1.4 or <0.6 with a significant difference (*p* < 0.05). 

## 3. Results

### 3.1. Regeneration and Establishment of Homozygous Lines

**Figure 1:**
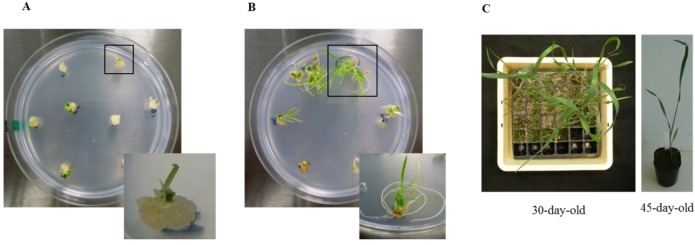
Regeneration of transgenic Bobwhite SH 98 26 after biolistic transformation of immature embryos with *Ubi:TaBWPR-1.2#13* (as a representative of both transgenes). (**A**) Shoot differentiation from calluses; (**B**) rooting of differentiated shoots; (**C**) transgenic wheat at 30 days (**left**) and 45 days (**right**).

Shoots regenerated six weeks after bombardment are shown in Figure 1A, and roots regenerated nine weeks after bombardment are shown in Figure 1B. Fewer plantlets were regenerated after bombardment with *Ubi:TaBWPR-1.2#2* than with *Ubi:TaBWPR-1.2#13* [38]. Plantlets were adapted to a room environment for seven days by removing paraffin with surgical tape and then transplanted into soil. Transgenic plants were established in soil in a plastic pocket tray after 30 days (Figure 1C, left) and subsequently transferred to plastic pots (Figure 1C, right) after 45 days of acclimatization.

Transgenic callus-derived plants (Figure 1A) were considered as T_0_ plants. The presence of transgenes was confirmed by DNA-PCR and RT-PCR analysis (Supplementary Figure 1, upper panels). T_1_ seeds were obtained from all T_0_ plants expressing the genes of interest. Sixteen seeds of each T_1_ plant were sown and segregation of transgenes in the leaf tip was confirmed by DNA-PCR and RT-PCR analysis (Supplementary Figure 1, lower panels). Nine out of twenty plants for *Ubi:TaBWPR-1.2#13* and seven out of 12 for *Ubi:TaBWPR-1.2#13* showed a Mendelian 3:1 ratio of transgene-positiveness. For each independent T_1_ plant, seven randomly selected transgene-positive plants and one transgene-negative plant were propagated to obtain T_2_ seeds and null-segregants. T_2_ seeds of all spikes from each individual positive T_1_ plant were bulked and further propagated to check homozygosity. Four independent, homozygous T_2_ lines were obtained for each, *Ubi:TaBWPR-1.2#2* (4, 11a, 11b', 13a) and *Ubi:TaBWPR-1.2#13* (2, 4a, 4b', 5b').

### 3.2. mRNA and Protein Levels in Ubi:TaBWPR-1.2 Transformants

**Figure 2:**
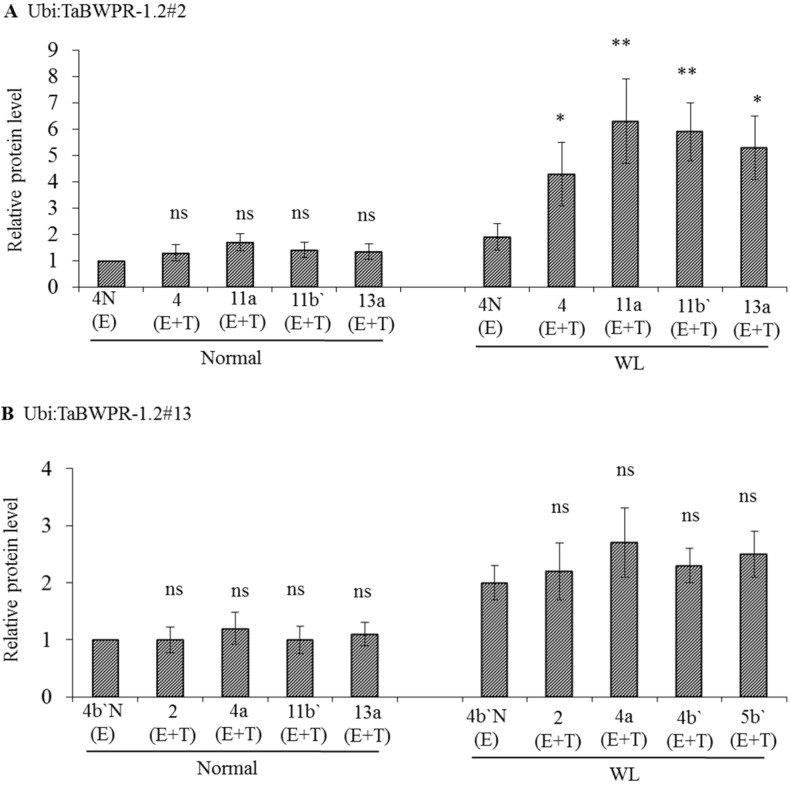
The levels of TaBWPR-1.2 proteins in the roots of homozygous transformants under control conditions and five days of waterlogging (WL). Immunoblotting was performed with anti-rice PR-1 antibody. (**A**) Four transgenic TaBWPR-1.2#2 lines; (**B**) four transgenic TaBWPR-1.2#13 lines. E, endogenous proteins; T, transgene proteins. The data are the means of three independent biological samples; error bars represent ±SEM. ns, not significant; * *p* < 0.05; ** *p* < 0.01 by a two-sample *t*-test.

To examine protein production in *Ubi:TaBWPR-1.2#2* and *Ubi:TaBWPR-1.2#13* transformants, we used immunoblotting of total protein from whole seminal roots of plants grown for five days under normal or waterlogged conditions. *Ubi:TaBWPR-1.2#2* transformants showed a slight, but not significant, increase in protein levels under normal conditions, but a significant increase under waterlogged conditions (Figure 2A). The increase in line L#11a was the largest among all lines. In *Ubi:TaBWPR-1.2#13* transformants, the protein level increased (but not significantly) under normal and waterlogged conditions; the increase was somewhat higher in L#4a than in other lines (Figure 2A).

**Figure 3:**
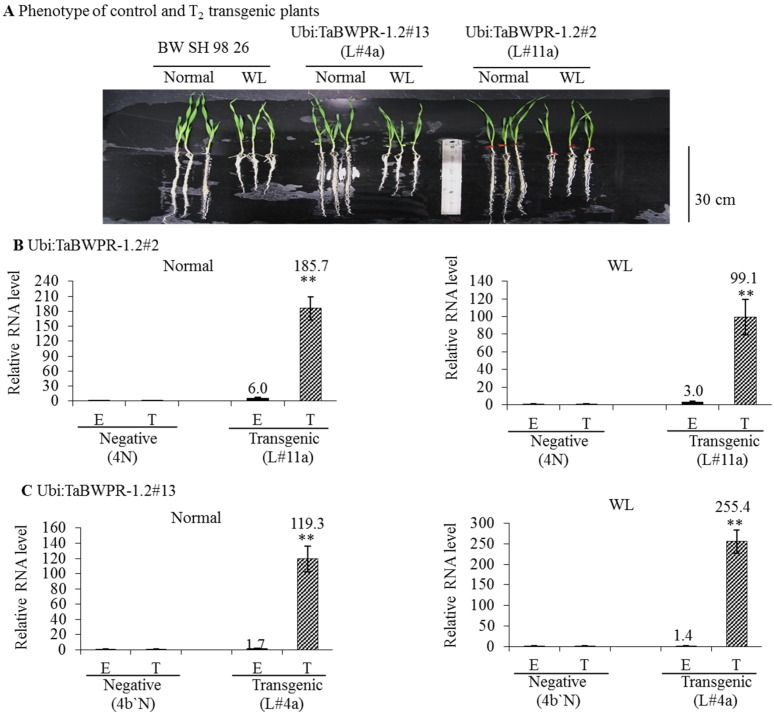
Expression of *TaBWPR-1.2* transgenes and respective endogenous genes inwheat roots under control conditions and after five days of waterlogging. (**A**) Phenotypes of control (Bobwhite SH 98 26) and the best transgenic lines (L#11a for *Ubi:TaBWPR-1.2#*2; L#4a for *TaBWPR-1.2#13*), showing no differences, except slightly longer roots under WL when compared to Bobwhite SH 98 26. *Ubi:TaBWPR-1.2#*2 L#11a (**B**) and *TaBWPR-1.2#13* L#4a (**C**) were compared with the respective negative lines (N). Transcript levels were normalized to an actin gene as an internal control. The relative mRNA levels of the E and T from negative plants were set to one. E, relative RNA levels of endogenous genes; T, relative RNA levels of transgenes. WL, waterlogged conditions. Data for endogenous and transgenes of transgenic seedlings are presented as solid black and hatched columns, respectively, whereas those for the negative controls are presented as white columns. The data are the means of three independent biological samples; error bars represent ±SEM. ** Significant differences (*p* < 0.01) by a two-sample *t*-test.

We compared the RNA levels of transgenes in the *Ubi:TaBWPR-1.2#2* (line L#11a) and *Ubi:TaBWPR-1.2#13* (line L#4a) transformants with the levels of corresponding endogenous RNA in the same samples by qRT-PCR. In L#11a, the level of the transgenic RNA was increased dramatically under both conditions, although the increase was less pronounced under waterlogged conditions (Figure 3B). The level of the endogenous RNA in this line was also increased and responded similarly to WL (Figure 3C). In L#4a, the level of the transgenic RNA was also dramatically increased, but the increase was higher under waterlogged than under normal conditions (Figure 3C). The level of the endogenous RNA was slightly elevated in L#4a under both conditions (Figure 3C). As shown in Figure 3A, when compared with Bobwhite SH 98 26, L#11a and L#4a transgenic lines showed only slightly longer roots under WL. L#11a and its null-segregant 4N were selected for proteome analysis.

### 3.3. Changes in Protein Levels in Seminal Roots of TaBWPR-1.2#2-Overexpressor Transgenic Plants

To gain insights into the physiological role of TaBWPR-1.2s in wheat root, we analyzed the total soluble proteome using gel-free proteomics technique in whole seminal roots of L#11a from *TaBWPR-1.2#2* and its null-segregant 4N grown under normal and waterlogged conditions. Under normal conditions, we detected two upregulated proteins (PR-1.17 and -1.14) and three downregulated proteins (PR, PR10 and unknown/proteasome subunit alpha type-3) (Table 1). When compared under waterlogged condition, we found four upregulated proteins (PR-1.6, ferredoxin precursor, elongation factor-2 (EF-2) and one unknown peptide (contig 2626) and one downregulated protein (PR) (Table 1). The upregulation of some PRs reconfirms that the *TaBWPR-1.2#2* transgene is at least translated into protein in L#11a. However, there are some other pathogenesis-related candidate proteins found to be up- and down-regulated in response to *TaBWPR-1.2#2* transgenic plants under both normal as well as waterlogged conditions. Results showed that there is a characteristic reverse tendency among the *PR-1* gene family. Silencing of tobacco *PR-1a* leads to silencing of other *PR-1* genes, but under certain treatments, some *PR-1* genes were upregulated in *PR-1a*-silenced tobacco plants [39]. There are 23 *TaPR-1* genes [19], and the expression of one of them may affect the expression of other *TaPR-1* genes. The functions of these new members of TaBWPR-1 proteins that are up- and down-regulated in L#11a are still unknown. In this study, a discussion was done on the possible application of the above four differentially expressed partner proteins of TaBWPR-1.2#2 in root physiology in response to soil WL.

**Table 1:** Changes in protein abundance in the seminal roots of the wheat transgenic line overexpressing *TaBWPR-1.2#2*.

	Protein Name	Accession No. ^a^	Organism	MP ^b^	Ratio ^c^	SD ^d^
	*Normal conditions (11a/4N)*					
1	Pathogenesis-related protein 1_17	F8S6U7	*T. aestivum*	4	3.0	0.45
2	Pathogenesis-related protein 1_14	F8S6U4	*T. aestivum*	2	1.6	0.5
3	Pathogenesis-related protein	H2KXF7	*T. aestivum*	4	0.44	0.02
4	Pathogenesis-related protein 10	B5B3P8	*T. aestivum*	4	0.46	0.08
5	Unknown Proteasome subunit alpha type-3	AK332255 *	*T. aestivum*	5	0.58	0.05
		ACN10361 *	*Salmo salar*			
	*WL conditions (11a/4N)*					
1	Pathogenesis-related protein 1_6	F8S6T6	*T. aestivum*	2	2.0	0.6
2	Ferredoxin precursor	Q8S3J5	*T. aestivum*	2	2.0	1.2
3	Elongation factor-2	Q9M7S5	*T. aestivum*	2	1.9	1.1
4	Unknown (contig 2626)	AK331943 *	*T. aestivum*	2	1.4	0.1
5	Pathogenesis-related protein	H2KXF7	*T. aestivum*	4	0.6	0.09

Protein hits were validated if identified with *p* < 0.05. ^a^ Accession numbers are from specific wheat databases (see main text) and from the NCBI database. * cDNA clones. ^b^ MP, the number of query-matched peptides (cutoff value: <3). ^c^ The ratio was calculated by dividing the protein level in transgenic wheat to that in wild-type wheat. ^d^ SD, standard deviation (*n* = 3).

## 4. Discussion

We produced homozygous transgenic *Ubi:TaBWPR-1.2* wheat and examined the RNA and proteins of seminal roots responsive to transgenes overexpression under normal and WL conditions. To the best of our knowledge, this is the first report of successful wheat transformation with *TaBWPR-1.2* constructs. Our transgenic *TaBWPR-1.2#2* line stably produced the RNA and protein of interest.

In comparison with the reported efficiency of biolistic transformation of wheat (1%) [40], the transformation efficiency in our study was low (approximately 0.2% for *Ubi:TaBWPR-1.2#2* and 0.4% for *Ubi:TaBWPR-1.2#13*), and it took us approximately two years to produce four homozygous lines for each transgene. The difference between the two transgenes might be due to the specific effects of these genes. We analyzed the expression of transgenes in various organs of four homozygous lines and one null-segregant from *Ubi:TaBWPR-1.2#13* in the absence of stress. The *Ubi:TaBWPR-1.2#13* transgene was ubiquitously expressed in germinating embryo and in all tested organs of 8- and 15-day-old seedlings (Supplementary Figure 2). We detected variations in both RNA expression (Figure 2 and Figure 3) and protein abundance (Supplementary Figure 2) among these lines. Studies in *Drosophila melanogaster* [41], *Saccharomyces cerevisiae* [42] and wheat [4] showed that the positions of the introduced genes on chromosomes may influence their expression. Thus, different insertion positions of the transgenes in the genome may have resulted in variations in their RNA expression. Under waterlogged conditions, the level of *TaBWPR-1.2#2* mRNA decreased (Figure 3B), whereas that of *TaBWPR-1.2#13* mRNA increased (Figure 3C). These differences in the stress response of transgene expression may also be caused by the insertion positions of the transgenes.

Line L#11a had the highest RNA expression and consistently produced the protein of interest under waterlogged conditions (Figure 3, Supplementary Figure 2). Although the effect of protein degradation and the difference in the detection of TaBWPR-1.2#2 and TaBWPR-1.2#13 by the extraction method and antibody used in this study cannot be excluded, we believe that *TaBWPR-1.2* mRNAs, particularly *TaBWPR-1.2#13* mRNA, are highly unstable. *TaBWPR-1.2* mRNAs reached its maximum at Day 1 after the onset of WL and then started to decline, which comes close to the baseline after Day 10 [26]. To bypass the adverse effects of constitutive overexpression, the early stress-responsive nature of *TaBWPR-1.2* genes requires suitable stress-inducible or root-specific promoters [6,18] and, thereby, sufficient activation for adequate translation in transgenic plants. The development of a WL-inducible promoter is urgently needed and is currently under way in our laboratory. Taking into account the difficulties in wheat transformation, L#11a and probably L#4a are good candidate lines with which to study the role of TaBWPR-1.2 proteins in wheat seminal roots. 

The level of PSMA3 was lower in line L#11a than in control plants under normal conditions, but not under WL. Proteasome-mediated proteolysis plays a key role in plant responses to several environmental stresses [43]. In soybean roots, accumulation of proteasome and COP9 signalosome proteins increases in response to flooding stress and returns close to baseline upon de-submergence [44]. Thus, it is suggested that PSMA3 in non-transgenic wheat increases upon WL. Based on the present result and previous information, the decreased amount of PSMA3 caused by TaBWPR-1.2#2 overproduction in L#11a may return close to baseline levels through the increase of its endogenous level upon WL, because transgene expression under normal and waterlogged conditions is the same as that of the *Ubi* promoter. This may be why PSMA3 is downregulated under normal conditions, but not under waterlogged conditions. Furthermore, Haque *et al.* [26] reported that the proteasome subunits did not increase in waterlogged wheat roots; the reason for the apparent discrepancy is that we previously used a more stringent threshold of two-fold differences in protein abundance. Although the mechanism of the PSMA3 decrease in L#11a is unclear and the decrease is moderate, it is indicated that it should be taken into account. It will be interesting to test whether TaBWPR-1.2#2 inhibits PSMA3 synthesis.

Ferredoxin (Fd) was increased in line L#11a under waterlogged conditions, but not under control conditions. Ferredoxins are iron-sulfur proteins that transfer electrons in a wide variety of metabolic reactions. In higher plants, distinct Fd isoforms are detected in photosynthetic and non-photosynthetic organs [45,46]. In non-photosynthetic root plastids, Fd-dependent enzymes need Fd reduced with NADPH (Fd:NADP^+^); one such enzyme is Fd:NADP^+^ oxidoreductase (FNR) [46,47]. Onda *et al.* [46] demonstrated that the interaction between root FNR and Fds was stronger than between leaf FNRs and Fds, which is crucial for efficient electron allocation and flux from NADH to Fd in the NADH-FNR-Fd cascade. Here, Fd increased in L#11a only under waterlogged conditions. It could be that a certain amount of PR-1.2 is needed to interact with Fd, which was not sufficient by overloaded TaBWPR-1.2#2 protein under control conditions, but together with elevated endogenous protein, the total TaBWPR-1.2#2 was sufficient under waterlogged conditions. We also found that Fd increases only under WL *vs.* control conditions in L#11, but is absent in the wild-type under WL *vs.* control conditions [38], suggesting that Fd is undetectable in the wild-type and responds only upon TaBWPR-1.2#2 expression. It is suggested that TaBWPR-1.2#2 may play an important role in a higher rate of electron flux in metabolic reactions mediated by Fd in wheat roots under the limited energy conditions caused by WL.

EF-2 is an essential protein catalyzing ribosomal translocation during protein synthesis [48], and EF accumulates in soybean under flooding stress [49]. Because protein synthesis needs to continue in plant roots under WL conditions [26], the increase in EF-2 in L#11a may regulate the synthesis of some proteins in wheat seminal roots. However, no EF-2 increase was found in either transgenic or non-transgenic plants compared between WL and control conditions (Supplementary Tables 2 and 3), suggesting that further studies are required to reconfirm that EF-2 is a responding protein to TaBWPR-1.2#2. Another potential responsive protein of TaBWPR-1.2#2 is encoded by contig 2626. Like Fd, this protein was present only in transgenic, but not in non-transgenic plants. Further studies are needed to elucidate the role of TaBWPR-1.2#2 in wheat roots, which involves the protein encoded by contig 2626. This study was performed in a phytotron chamber under mild WL stress conditions; hence the identification of more TaBWPR-1.2-responsive proteins can be expected under much more severe stress conditions, such as waterlogged conditions in a greenhouse [26]. These results suggest that TaBWPR-1.2#2 appears to be an inhibitor of the proteasome under normal conditions and an inducer of Fd and EF-2 under WL, and TaBWPR-1.2#2 might be a potential candidate root protein that mitigates the effects of WL. 

## 5. Conclusions

We developed transgenic wheat lines overexpressing two *TaBWPR-1.2* genes and obtained some evidence regarding the physiological pathways possibly affected by TaBWPR-1.2#2 in wheat roots under WL. Further studies are needed to develop transgenic wheat lines expressing *TaBWPR-1.2* genes under the control of root-specific or WL-inducible promoters to examine the phenotypic responses under more natural waterlogged conditions.

## Supplementary Materials

Supplementary File 1
