# Supplementary material for: Quantitative Proteomics of the Root of Transgenic Wheat Expressing TaBWPR-1.2 Genes in Response to Waterlogging
Source: Proteomes. 2014 Nov 4;2(4):485–500. doi: 10.3390/proteomes2040485 (PMC5302695; doi:10.3390/proteomes2040485)
Supplement: Supplementary File 1 [file proteomes-02-00485-s001.pdf]

# Supplementary Material

**Figure S1.** PCR analysis of the presence of transgenes and mRNA expression in $T_0$ and $T_1$ generations of wheat plants transformed with *TaBWPR-1.2* constructs. $T_0$: (**top**) analysis of leaf genomic DNA of 20 (for *Ubi:TaBWPR-1.2#2*) and 12 (for *Ubi:TaBWPR-1.2#13*) putative $T_0$ lines; (**bottom**) analysis of leaf mRNA from the same plants. $T_1$: to confirm the 3:1 segregation ratio, 16 plants were examined from each of the *Ubi:TaBWPR-1.2#2* and *Ubi:TaBWPR-1.2#13* lines. Seven DNA-positive plants and one DNA-negative plant were tested by RT-PCR. For each construct, four representative independent transgenic lines that were isolated as homozygous $T_2$ are shown. P, positive plasmid controls; N, non-transgenic control.

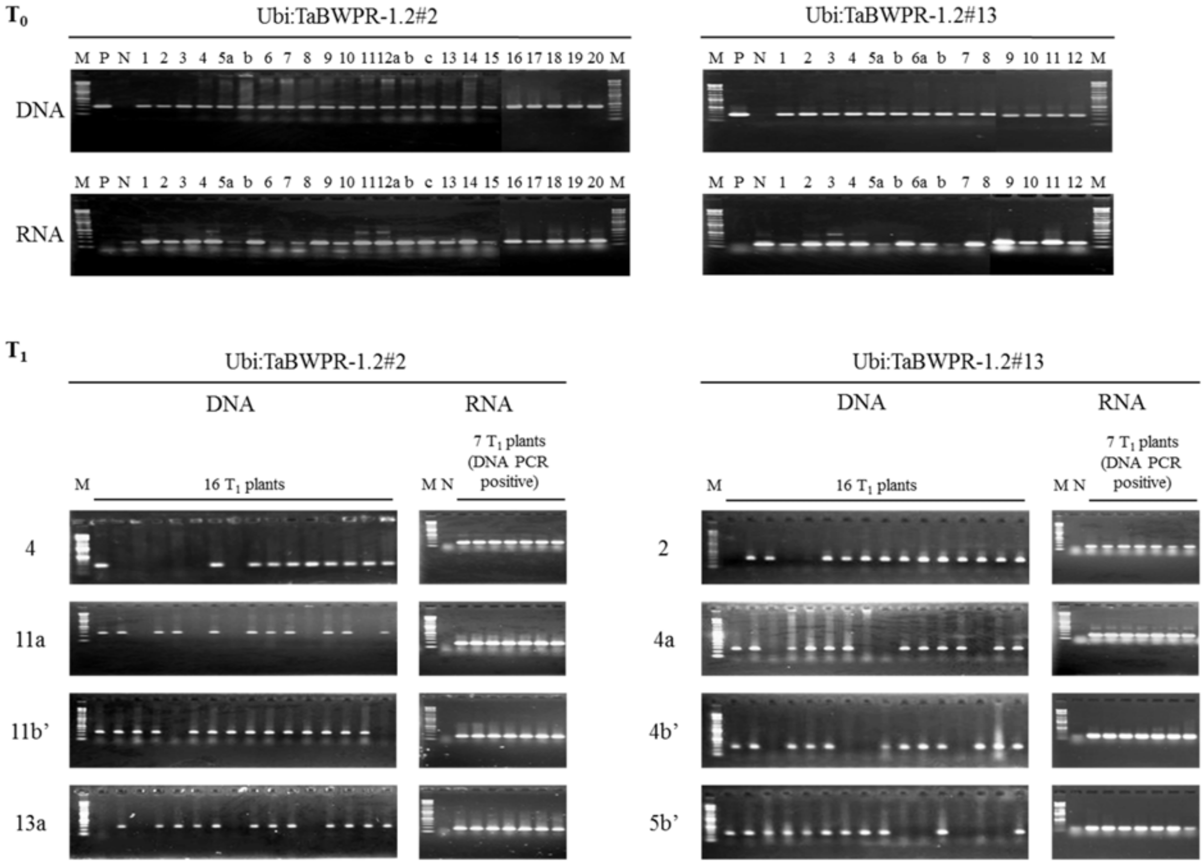

**Figure S2.** Organ-specific expression of *Ubi:TaBWPR-1.2#13* (as a representative of both transgenes) detected by RT-PCR. (**Top**) A four-day-old germinating embryo. (**Middle**) Leaf, root base (1 cm), middle of root (3–5 cm) and root tip (1 cm) of an eight-day-old seedling. (**Bottom**, **left**) Whole leaf and root of a 15-day-old seedling grown in a pot. (**Bottom**, **right**) Spikes (before anthesis) of 90-day-old plants grown in pots. N, respective null-segregant; P, positive plasmid controls.

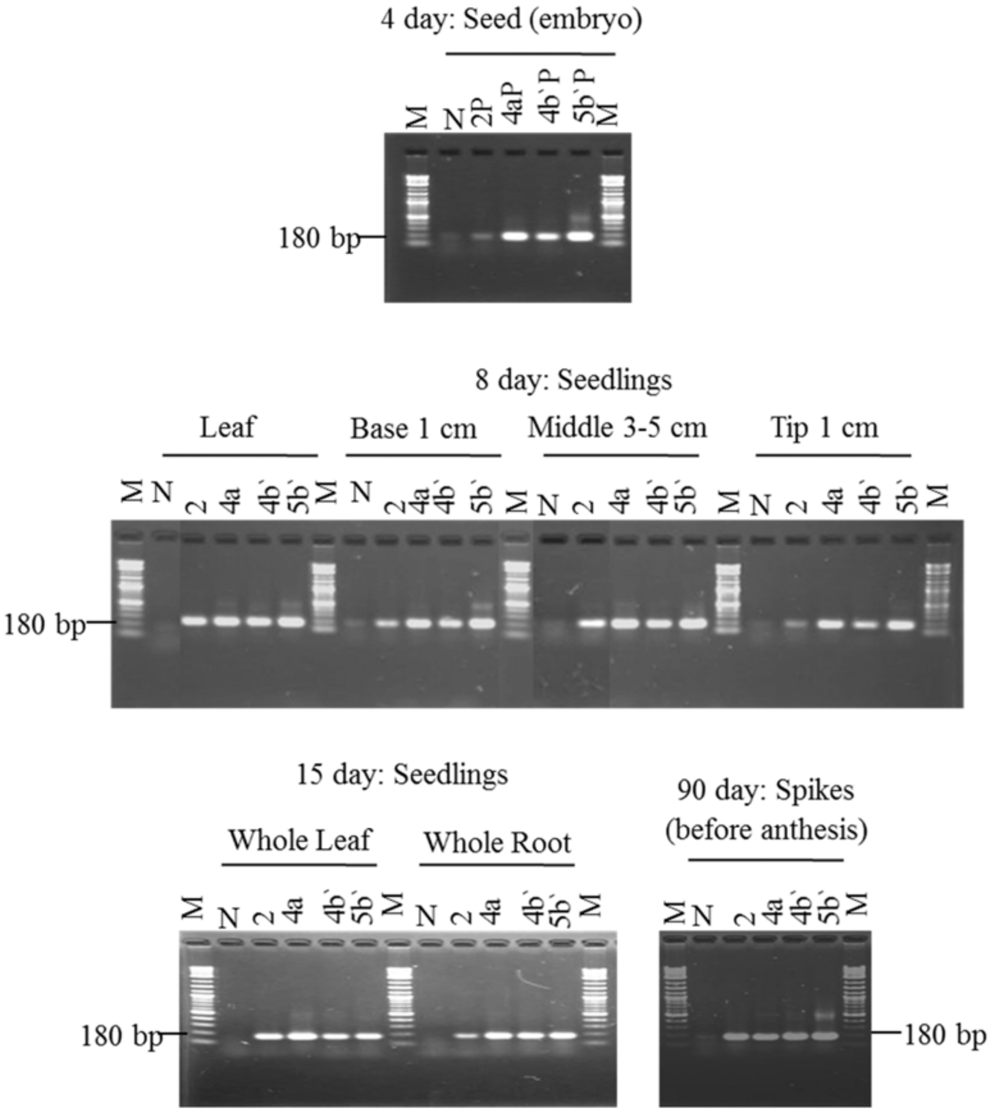

**Figure S3.** The levels of TaBWPR-1.2 proteins in the roots of homozygous transformants under control conditions and five days of WL. Immunoblotting was performed with anti-rice PR-1 antibody. The molecular weight of TaBWPR-1.2 is shown.

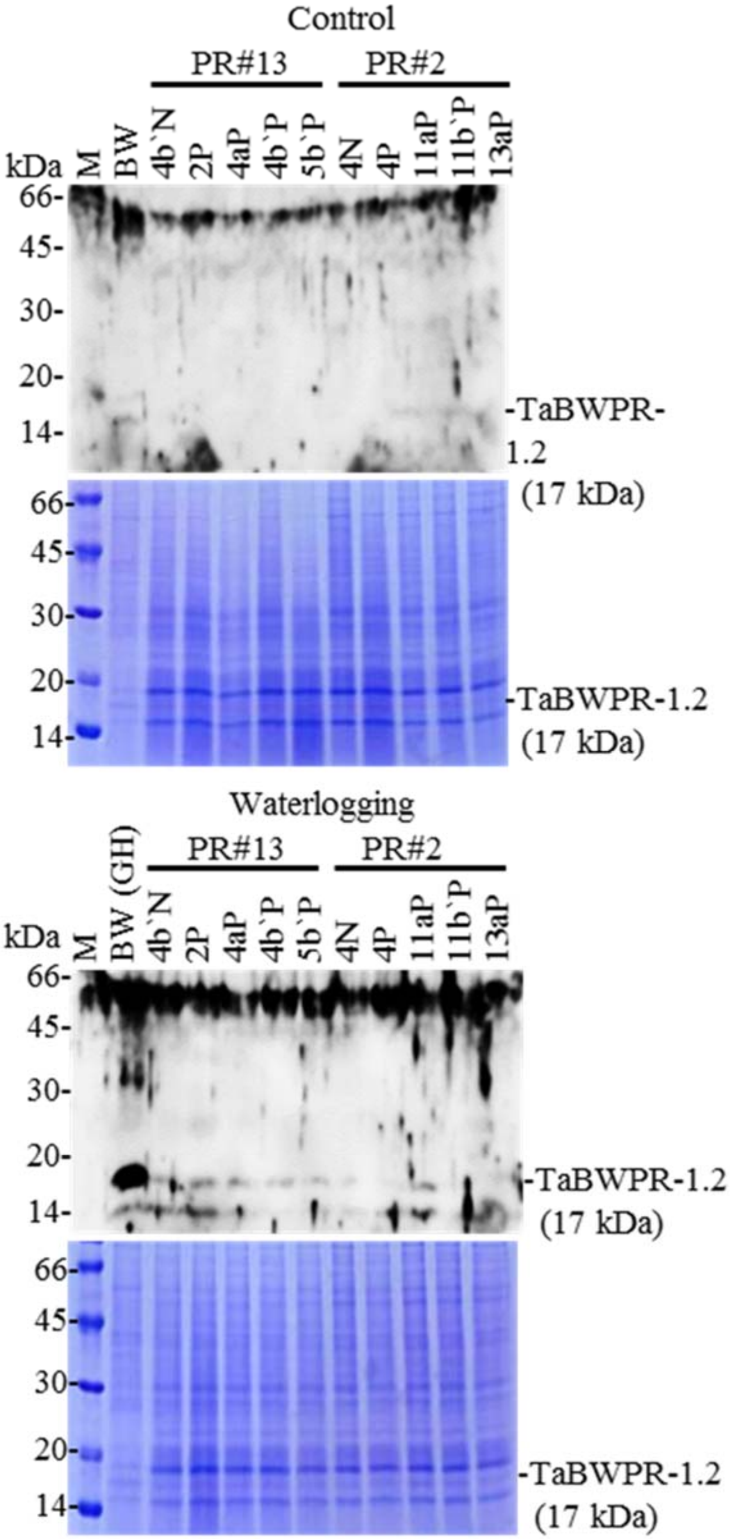

**Table S1.** Composition of media.

| Medium Name | Composition |
|---|---|
| Callus induction: 0.2 M mannitol (CI-0.2Man) | MS salts and vitamins [1], 0.2 M mannitol, 1 mg·L$^{-1}$ thiamin-HCl, 1.95 g·L$^{-1}$ MES, 3.5 mM Gln, 1 mM Pro, 1 mM Asn, 100 mg·L$^{-1}$ casein hydrolysate, pH 5.8, 2 g·L$^{-1}$ Phytagel (Sigma), autoclaved. Picloram (2 mg·L$^{-1}$), 2 mg·L$^{-1}$ 2,4-dichlorophenoxyacetic acid (2,4-D), 100 mg·L$^{-1}$ ascorbic acid, and 150 g·L$^{-1}$ maltose added after autoclaving |
| Callus maintenance: 3 mg·L$^{-1}$ phosphinothricin (CM-3P) | Same as CI-0.2Man, but no mannitol, and 0.5 mg·L$^{-1}$ 2,4-D. Add 3 mg·L$^{-1}$ phosphinothricin (PPT) after autoclaving. |
| Shoot growth: 1 mg·L$^{-1}$ PPT (SG-1P) | Macrosalts (1.4 g·L$^{-1}$ $KNO_3$, 300 mg·L$^{-1}$ $NH_4NO_3$, 200 mg·L$^{-1}$ $KH_2PO_4$, 450 mg·L$^{-1}$ $CaCl_2·2H_2O$, 350 mg·L$^{-1}$ $MgSO_4·7H_2O$); microsalts (40 mg·L$^{-1}$ Fe·Na·EDTA, 11.2 mg·L$^{-1}$ $MnSO_4·5H_2O$, 5 mg·L$^{-1}$ $H_3BO_3$, 7.5 mg·L$^{-1}$ $ZnSO_4·7H_2O$, 0.75 mg·L$^{-1}$ KI, 0.25 mg·L$^{-1}$ $Na_2MoO_4·7H_2O$, 0.025 mg·L$^{-1}$ $CuSO_4·5H_2O$, 0.025 mg·L$^{-1}$ $CoCl_2·6H_2O$); 30 g·L$^{-1}$ maltose, 200 mg·L$^{-1}$ myo-inositol, pH 5.7, 5 g·L$^{-1}$ agar, autoclaved. Zeatin (5 mg·L$^{-1}$), 0.1 mg·L$^{-1}$ 2,4-D, vitamins (10 mg·L$^{-1}$ thiamin-HCl, 1 mg·L$^{-1}$ pyridoxine-HCl, 1 mg·L$^{-1}$ nicotinic acid, 1 mg·L$^{-1}$ Ca-pantothenate, 1 mg·L$^{-1}$ ascorbic acid) and 1 mg·L$^{-1}$ PPT added after autoclaving. |
| Root growth: 3 mg·L$^{-1}$ PPT (RG-3P) | Same as SG-1P, but no zeatin or 2,4-D and 3 mg·L$^{-1}$ PPT |

**Table S2.** The list of proteins identified in seminal roots of the wheat transgenic line and the wild-type under control condition.

| PROTEIN ID | DESCRIPTION | Matched Peptides | FRAMES | HITS | Ratio | SD | *p*-Value |
|---|---|---|---|---|---|---|---|
| Increase | | | | | | | |
| UniRef100_F8S6U7 | Pathogenesis_related protein 1_17 *n* = 1 Tax = *Triticum aestivum* RepID = F8S6U7_WHEAT | 4 | 7 | 62 | 2.88 | 0.46 | 0.00 |
| UniRef100_F8S6U4 | Pathogenesis_related protein 1_14 *n* = 1 Tax = *Triticum aestivum* RepID = F8S6U4_WHEAT | 2 | 3 | 15 | 1.60 | 0.57 | 0.00 |
| RFL_Contig3922 | _pep__1:127_2472 | 4 | 4 | 6 | 1.33 | 0.22 | 0.03 |
| gi_257637546_emb_CBD24658.1_ | unnamed protein product (*Triticum aestivum*) | 2 | 3 | 7 | 1.30 | 0.21 | 0.01 |
| RFL_Contig60 | _pep__1:127_2376 | 2 | 2 | 3 | 1.28 | 0.15 | 0.01 |
| UniRef100_Q9ZR95 | Gamma_type tonoplast intrinsic protein *n* = 1 Tax = *Triticum aestivum* RepID = Q9ZR95_WHEAT | 2 | 3 | 25 | 1.26 | 0.26 | 0.00 |
| UniRef100_Q45NB5 | Glutamine synthetase *n* = 1 Tax = *Triticum aestivum* RepID = Q45NB5_WHEAT | 2 | 3 | 17 | 1.26 | 0.28 | 0.03 |
| UniRef100_D2KZ10 | Alanine_glyoxylate aminotransferase *n* = 1 Tax = *Triticum aestivum* RepID = D2KZ10_WHEAT | 3 | 3 | 10 | 1.25 | 0.12 | 0.02 |
| RFL_Contig5231 | _pep__1:85_540 | 3 | 3 | 17 | 1.22 | 0.21 | 0.00 |
| RFL_Contig267 | _pep__1:103_981 | 3 | 3 | 7 | 1.22 | 0.11 | 0.00 |
| RFL_Contig168 | _pep__3:78_440 | 2 | 2 | 15 | 1.21 | 0.17 | 0.00 |
| UniRef100_Q9FXQ8 | TaWIN2 *n* = 1 Tax = *Triticum aestivum* RepID = Q9FXQ8_WHEAT | 6 | 7 | 17 | 1.20 | 0.11 | 0.00 |
| RFL_Contig1992 | _pep__2:74_1693 | 5 | 5 | 19 | 1.19 | 0.19 | 0.04 |
| RFL_Contig5189 | _pep__3:90_845 | 2 | 2 | 14 | 1.19 | 0.17 | 0.00 |
| UniRef100_Q401N6 | Aspartic proteinase *n* = 1 Tax = *Triticum aestivum* RepID = Q401N6_WHEAT | 4 | 4 | 32 | 1.19 | 0.11 | 0.00 |
| UniRef100_H9CWE9 | 12_oxo_phytodienoic acid reductase *n* = 1 Tax = *Triticum aestivum* RepID = H9CWE9_WHEAT | 2 | 2 | 15 | 1.19 | 0.09 | 0.00 |
| UniRef100_A9P8I4 | Predicted protein *n* = 6 Tax = Magnoliophyta RepID = A9P8I4_POPTR | 2 | 2 | 23 | 1.18 | 0.07 | 0.00 |
| gi_227473231_emb_CAY33013.1_ | unnamed protein product (*Triticum aestivum*) | 7 | 8 | 39 | 1.17 | 0.10 | 0.00 |
| UniRef100_B4F6E5 | Root peroxidase *n* = 1 Tax = *Triticum aestivum* RepID = B4F6E5_WHEAT | 6 | 6 | 14 | 1.17 | 0.14 | 0.00 |
| UniRef100_B4F6F2 | Root peroxidase *n* = 1 Tax = *Triticum aestivum* RepID = B4F6F2_WHEAT | 7 | 7 | 15 | 1.16 | 0.13 | 0.00 |
| UniRef100_Q84MJ5 | Methylmalonate semialdehyde dehydrogenase (Fragment) *n* = 1 Tax = *Triticum aestivum* RepID = Q84MJ5_WHEAT | 5 | 7 | 42 | 1.16 | 0.10 | 0.05 |
| RFL_Contig4308 | _pep__3:60_830 | 3 | 3 | 11 | 1.15 | 0.10 | 0.03 |
| UniRef100_O81331 | Vacuolar invertase (Fragment) *n* = 1 Tax = *Triticum aestivum* RepID = O81331_WHEAT | 5 | 6 | 33 | 1.14 | 0.25 | 0.01 |
| RFL_Contig5913 | _pep__1:166_957 | 4 | 6 | 36 | 1.14 | 0.13 | 0.00 |
| UniRef100_Q7X729 | Acidic ribosomal protein P2 (Fragment) *n* = 1 Tax = *Triticum aestivum* RepID = Q7X729_WHEAT | 2 | 4 | 30 | 1.14 | 0.09 | 0.00 |
| UniRef100_Q75QN9 | Cold shock domain protein 2 *n* = 1 Tax = *Triticum aestivum* RepID = Q75QN9_WHEAT | 4 | 4 | 32 | 1.13 | 0.07 | 0.00 |
| UniRef100_A3FKE5 | Superoxide dismutase (Fragment) *n* = 1 Tax = *Triticum aestivum* RepID = A3FKE5_WHEAT | 2 | 2 | 8 | 1.12 | 0.19 | 0.01 |

**Table S2.** *Cont.*

| PROTEIN ID | DESCRIPTION | Matched Peptides | FRAMES | HITS | Ratio | SD | *p*-Value |
|---|---|---|---|---|---|---|---|
| RFL_Contig3422 | _pep__2:98_514 | 2 | 2 | 22 | 1.12 | 0.13 | 0.00 |
| RFL_Contig2381 | _pep__2:65_808 | 2 | 4 | 20 | 1.12 | 0.11 | 0.00 |
| RFL_Contig1475 | _pep__1:52_537 | 2 | 2 | 3 | 1.11 | 0.13 | 0.04 |
| gi_218381856_emb_CAV24596.1_ | unnamed protein product (*Triticum aestivum*) | 2 | 3 | 30 | 1.11 | 0.09 | 0.00 |
| UniRef100_O24396 | Adenylosuccinate synthetase_ chloroplastic (Fragment) *n* = 1 Tax = *Triticum aestivum* RepID = PURA_WHEAT | 3 | 3 | 18 | 1.11 | 0.08 | 0.02 |
| UniRef100_A5JPR2 | Peroxisomal ascorbate peroxidase *n* = 2 Tax = Triticeae RepID = A5JPR2_WHEAT | 4 | 4 | 26 | 1.11 | 0.06 | 0.00 |
| UniRef100_Q5G1T9 | Gamma_glutamylcysteine synthetase *n* = 1 Tax = *Triticum aestivum* RepID = Q5G1T9_WHEAT | 4 | 5 | 29 | 1.11 | 0.11 | 0.01 |
| UniRef100_B4F6E6 | Root peroxidase *n* = 1 Tax = *Triticum aestivum* RepID = B4F6E6_WHEAT | 9 | 9 | 65 | 1.10 | 0.11 | 0.00 |
| UniRef100_B4F6E7 | Root peroxidase *n* = 1 Tax = *Triticum aestivum* RepID = B4F6E7_WHEAT | 9 | 10 | 91 | 1.10 | 0.10 | 0.00 |
| RFL_Contig3447 | _pep__3:195_1160 | 2 | 2 | 6 | 1.10 | 0.07 | 0.01 |
| UniRef100_I0AW27 | Mitochondrial manganese superoxide dismutase (Fragment) *n* = 1 Tax = *Triticum aestivum* RepID = I0AW27_WHEAT | 3 | 3 | 5 | 1.09 | 0.17 | 0.02 |
| UniRef100_Q7X9L9 | QM (Fragment) *n* = 1 Tax = *Triticum aestivum* RepID = Q7X9L9_WHEAT | 2 | 2 | 17 | 1.09 | 0.33 | 0.01 |
| RFL_Contig2937 | _pep__3:102_1607 | 5 | 6 | 27 | 1.09 | 0.09 | 0.03 |
| UniRef100_G1FUU8 | Superoxide dismutase *n* = 1 Tax = *Triticum aestivum* RepID = G1FUU8_WHEAT | 3 | 3 | 19 | 1.08 | 0.11 | 0.02 |
| gi_257667483_emb_CBD31968.1_ | unnamed protein product (*Triticum aestivum*) | 4 | 5 | 36 | 1.08 | 0.08 | 0.00 |
| RFL_Contig3430 | _pep__3:291_1733 | 5 | 5 | 22 | 1.08 | 0.06 | 0.04 |
| UniRef100_Q7XYD5 | Acidic ribosomal protein (Fragment) *n* = 1 Tax = *Triticum aestivum* RepID = Q7XYD5_WHEAT | 4 | 6 | 68 | 1.08 | 0.08 | 0.00 |
| UniRef100_Q2QKB2 | MRNA transport factor *n* = 1 Tax = *Triticum aestivum* RepID = Q2QKB2_WHEAT | 2 | 2 | 13 | 1.08 | 0.13 | 0.00 |
| UniRef100_D8L9A5 | Putative PDI_like protein *n* = 1 Tax = *Triticum aestivum* RepID = D8L9A5_WHEAT | 5 | 5 | 20 | 1.08 | 0.05 | 0.00 |
| RFL_Contig3855 | _pep__3:126_1601 | 5 | 6 | 44 | 1.07 | 0.06 | 0.00 |
| RFL_Contig3939 | _pep__2:125_1702 | 8 | 10 | 53 | 1.07 | 0.08 | 0.01 |
| gi_296524610_emb_CBM36960.1_ | unnamed protein product (*Triticum aestivum*) | 7 | 8 | 59 | 1.06 | 0.05 | 0.00 |
| gi_296525620_emb_CBM37262.1_ | unnamed protein product (*Triticum aestivum*) | 6 | 6 | 43 | 1.06 | 0.10 | 0.01 |
| UniRef100_C6K7G3 | Lipoxygenase *n* = 1 Tax = *Triticum aestivum* RepID = C6K7G3_WHEAT | 7 | 8 | 22 | 1.06 | 0.05 | 0.00 |
| RFL_Contig2671 | _pep__1:118_1575 | 8 | 8 | 66 | 1.06 | 0.07 | 0.01 |
| RFL_Contig825 | _pep__1:61_3579 | 9 | 9 | 40 | 1.06 | 0.05 | 0.00 |
| gi_219890652_emb_CAW94684.1_ | unnamed protein product (*Triticum aestivum*) | 10 | 13 | 72 | 1.05 | 0.04 | 0.00 |
| gi_227289532_emb_CAY02762.1_ | unnamed protein product (*Triticum aestivum*) | 2 | 2 | 10 | 1.05 | 0.06 | 0.00 |

**Table S2.** *Cont.*

| PROTEIN ID | DESCRIPTION | Matched Peptides | FRAMES | HITS | Ratio | SD | *p*-Value |
|---|---|---|---|---|---|---|---|
| gi_219890650_emb_CAW94682.1_ | unnamed protein product (*Triticum aestivum*) | 6 | 7 | 28 | 1.05 | 0.05 | 0.00 |
| RFL_Contig2744 | _pep__1:109_1791 | 4 | 4 | 13 | 1.05 | 0.14 | 0.00 |
| gi_219914284_emb_CAW74911.1_ | unnamed protein product (*Triticum aestivum*) | 7 | 8 | 48 | 1.05 | 0.11 | 0.01 |
| UniRef100_Q8VYX1 | Phosphoethanolamine methyltransferase *n* = 1 Tax = *Triticum aestivum* RepID = Q8VYX1_WHEAT | 9 | 10 | 62 | 1.04 | 0.04 | 0.05 |
| UniRef100_G3E8E1 | Aquaporin 7 *n* = 1 Tax = *Triticum aestivum* RepID = G3E8E1_WHEAT | 5 | 7 | 70 | 1.04 | 0.07 | 0.00 |
| RFL_Contig2753 | _pep__3:177_1784 | 6 | 7 | 30 | 1.03 | 0.04 | 0.05 |
| UniRef100_Q9M7C4 | Plasma membrane intrinsic protein 1 *n* = 1 Tax = *Triticum aestivum* RepID = Q9M7C4_WHEAT | 2 | 3 | 31 | 1.03 | 0.14 | 0.00 |
| RFL_Contig2735 | _pep__1:76_2670 | 11 | 14 | 93 | 1.03 | 0.04 | 0.00 |
| UniRef100_P55313 | Catalase *n* = 1 Tax = *Triticum aestivum* RepID = CATA2_WHEAT | 8 | 10 | 58 | 1.03 | 0.04 | 0.04 |
| RFL_Contig3291 | _pep__3:81_1841 | 10 | 13 | 96 | 1.03 | 0.05 | 0.00 |
| UniRef100_C7C4X1 | Glyceraldehyde_3_phosphate dehydrogenase *n* = 1 Tax = *Triticum aestivum* RepID = C7C4X1_WHEAT | 10 | 21 | 184 | 1.03 | 0.04 | 0.00 |
| RFL_Contig1280 | _pep__3:195_1787 | 3 | 4 | 36 | 1.03 | 0.02 | 0.01 |
| RFL_Contig3876 | _pep__2:95_1588 | 5 | 6 | 15 | 1.03 | 0.10 | 0.00 |
| RFL_Contig3093 | _pep__1:85_957 | 3 | 4 | 7 | 1.03 | 0.13 | 0.00 |
| RFL_Contig3171 | _pep__2:98_1981 | 6 | 6 | 20 | 1.02 | 0.07 | 0.00 |
| gi_315113253_pdb_3IZR_H | Chain H_ Localization Of The Large Subunit Ribosomal Proteins Into A 5.5 A Cryo_Em Map of *Triticum aestivum* Translating 80s Ribosome | 5 | 5 | 13 | 1.02 | 0.04 | 0.00 |
| RFL_Contig2987 | _pep__2:68_565 | 5 | 5 | 31 | 1.02 | 0.14 | 0.02 |
| gi_227483057_emb_CAY37266.1_ | unnamed protein product (*Triticum aestivum*) | 11 | 14 | 89 | 1.02 | 0.03 | 0.00 |
| RFL_Contig6066 | _pep__3:69_2492 | 13 | 13 | 95 | 1.02 | 0.04 | 0.00 |
| UniRef100_P46524 | Dehydrin COR410 *n* = 2 Tax = *Triticum aestivum* RepID = CO410_WHEAT | 3 | 4 | 26 | 1.01 | 0.11 | 0.00 |
| RFL_Contig2913 | _pep__2:56_544 | 4 | 5 | 31 | 1.01 | 0.03 | 0.00 |
| RFL_Contig3927 | _pep__3:123_1715 | 5 | 5 | 30 | 1.01 | 0.05 | 0.00 |
| RFL_Contig5059 | _pep__1:97_1608 | 7 | 8 | 47 | 1.01 | 0.05 | 0.00 |
| UniRef100_C5H4Q0 | Class III peroxidase *n* = 1 Tax = *Triticum aestivum* RepID = C5H4Q0_WHEAT | 5 | 6 | 38 | 1.01 | 0.09 | 0.01 |
| RFL_Contig308 | _pep__1:73_1512 | 10 | 15 | 113 | 1.01 | 0.06 | 0.01 |
| UniRef100_D8L9J3 | Dolichyl_diphosphooligosaccharide__protein glycosyltransferase_ putative_ expressed *n* = 1 Tax = *Triticum aestivum* RepID = D8L9J3_WHEAT | 11 | 12 | 64 | 1.01 | 0.04 | 0.01 |

**Table S2.** *Cont.*

| PROTEIN ID | DESCRIPTION | Matched Peptides | FRAMES | HITS | Ratio | SD | *p*-Value |
|---|---|---|---|---|---|---|---|
| Decrease | | | | | | | |
| UniRef100_H2KXF7 | Pathogenesis_related protein *n* = 1 Tax = *Triticum aestivum* RepID = H2KXF7_WHEAT | 4 | 5 | 16 | 0.44 | 0.08 | 0.00 |
| UniRef100_B5B3P8 | Pathogenesis related protein 10 *n* = 2 Tax = commelinids RepID = B5B3P8_WHEAT | 4 | 4 | 16 | 0.47 | 0.08 | 0.00 |
| RFL_Contig2907 | _pep__2:905_1681 | 5 | 5 | 11 | 0.58 | 0.05 | 0.00 |
| RFL_Contig2814 | _pep__2:74_1759 | 6 | 6 | 19 | 0.59 | 0.03 | 0.00 |
| UniRef100_Q41629 | ADP_ATP carrier protein 1_ mitochondrial *n* = 1 Tax = *Triticum aestivum* RepID = ADT1_WHEAT | 7 | 10 | 68 | 0.60 | 0.03 | 0.00 |
| UniRef100_Q43223 | Sucrose synthase type 2 *n* = 2 Tax = *Triticum aestivum* RepID = Q43223_WHEAT | 6 | 8 | 40 | 0.69 | 0.07 | 0.00 |
| RFL_Contig4468 | _pep__3:39_2093 | 2 | 2 | 15 | 0.70 | 0.23 | 0.01 |
| RFL_Contig154 | _pep__3:54_440 | 2 | 3 | 18 | 0.73 | 0.18 | 0.00 |
| UniRef100_Q5I5K7 | Small GTP_binding protein *n* = 1 Tax = *Triticum aestivum* RepID = Q5I5K7_WHEAT | 8 | 9 | 40 | 0.74 | 0.07 | 0.00 |
| UniRef100_I1HEK5 | Uncharacterized protein *n* = 2 Tax = Pooideae RepID = I1HEK5_BRADI | 2 | 2 | 17 | 0.76 | 0.07 | 0.00 |
| UniRef100_A4K4Z0 | Alpha tubulin_5B *n* = 1 Tax = *Triticum aestivum* RepID = A4K4Z0_WHEAT | 4 | 7 | 68 | 0.77 | 0.05 | 0.00 |
| UniRef100_Q8LGQ9 | Betaine_aldehyde dehydrogenase *n* = 1 Tax = *Triticum aestivum* RepID = Q8LGQ9_WHEAT | 6 | 6 | 38 | 0.77 | 0.06 | 0.00 |
| UniRef100_A2WN93 | Calmodulin_1 *n* = 18 Tax = Magnoliophyta RepID = CALM1_ORYSI | 3 | 3 | 6 | 0.78 | 0.06 | 0.00 |
| UniRef100_P04464 | Calmodulin *n* = 1 Tax = *Triticum aestivum* RepID = CALM_WHEAT | 3 | 3 | 34 | 0.78 | 0.06 | 0.00 |
| UniRef100_Q8RW03 | Glutathione transferase *n* = 1 Tax = *Triticum aestivum* RepID = Q8RW03_WHEAT | 4 | 4 | 8 | 0.79 | 0.04 | 0.00 |
| RFL_Contig2611 | _pep__1:163_2028 | 4 | 4 | 19 | 0.79 | 0.05 | 0.00 |
| UniRef100_Q8RW02 | Glutathione transferase *n* = 1 Tax = *Triticum aestivum* RepID = Q8RW02_WHEAT | 5 | 6 | 37 | 0.79 | 0.04 | 0.00 |
| UniRef100_Q8L808 | Putative cytochrome c oxidase subunit *n* = 1 Tax = *Triticum aestivum* RepID = Q8L808_WHEAT | 2 | 2 | 10 | 0.79 | 0.12 | 0.00 |
| RFL_Contig3301 | _pep__1:73_1101 | 3 | 3 | 12 | 0.80 | 0.11 | 0.01 |
| RFL_Contig3551 | _pep__3:141_1922 | 4 | 4 | 17 | 0.81 | 0.10 | 0.00 |
| RFL_Contig3524 | _pep__2:224_3232 | 4 | 4 | 13 | 0.81 | 0.09 | 0.00 |
| UniRef100_Q8RW01 | Glutathione transferase *n* = 1 Tax = *Triticum aestivum* RepID = Q8RW01_WHEAT | 2 | 2 | 10 | 0.81 | 0.13 | 0.00 |
| gi_315113258_pdb_3IZR_Q | Chain Q_ Localization Of The Large Subunit Ribosomal Proteins Into A 5.5 A Cryo_Em Map of *Triticum aestivum* Translating 80s Ribosome | 3 | 3 | 10 | 0.82 | 0.06 | 0.00 |
| UniRef100_Q9ZR33 | Glycosyltransferase 75 *n* = 1 Tax = *Triticum aestivum* RepID = Q9ZR33_WHEAT | 11 | 13 | 118 | 0.82 | 0.05 | 0.00 |
| RFL_Contig3262 | _pep__3:150_1172 | 3 | 4 | 14 | 0.82 | 0.09 | 0.00 |

**Table S2.** *Cont.*

| PROTEIN ID | DESCRIPTION | Matched Peptides | FRAMES | HITS | Ratio | SD | *p*-Value |
|---|---|---|---|---|---|---|---|
| gi_257669840_emb_CBD25095.1_ | unnamed protein product (*Triticum aestivum*) | 3 | 4 | 41 | 0.83 | 0.07 | 0.00 |
| RFL_Contig3224 | _pep__2:101_2215 | 20 | 22 | 228 | 0.83 | 0.04 | 0.00 |
| RFL_Contig608 | _pep__1:118_1374 | 2 | 2 | 7 | 0.83 | 0.19 | 0.02 |
| UniRef100_F6H5Z7 | Putative uncharacterized protein *n* = 11 Tax = Viridiplantae RepID = F6H5Z7_VITVI | 3 | 9 | 82 | 0.84 | 0.08 | 0.00 |
| RFL_Contig3004 | _pep__1:253_1263 | 2 | 2 | 11 | 0.84 | 0.14 | 0.00 |
| RFL_Contig4661 | _pep__3:114_1046 | 5 | 5 | 23 | 0.84 | 0.07 | 0.00 |
| UniRef100_Q6IY71 | Mitochondrial ATP synthase *n* = 1 Tax = *Triticum aestivum* RepID = Q6IY71_WHEAT | 5 | 6 | 32 | 0.84 | 0.11 | 0.01 |
| UniRef100_Q43199 | Adenine phosphoribosyltransferase 1 *n* = 1 Tax = *Triticum aestivum* RepID = APT1_WHEAT | 5 | 6 | 32 | 0.85 | 0.07 | 0.01 |
| UniRef100_G9HXG9 | 60S ribosomal protein L5 *n* = 1 Tax = *Triticum aestivum* RepID = G9HXG9_WHEAT | 3 | 5 | 50 | 0.85 | 0.07 | 0.00 |
| UniRef100_Q08G39 | Ribosomal protein L3 *n* = 1 Tax = *Triticum aestivum* RepID = Q08G39_WHEAT | 6 | 10 | 56 | 0.86 | 0.05 | 0.00 |
| RFL_Contig3052 | _pep__3:75_488 | 2 | 2 | 12 | 0.86 | 0.17 | 0.02 |
| UniRef100_Q5I7K2 | Ribosomal protein S7 *n* = 1 Tax = *Triticum aestivum* RepID = Q5I7K2_WHEAT | 6 | 8 | 33 | 0.86 | 0.12 | 0.00 |
| UniRef100_Q1W692 | Calcium_dependent protein kinase *n* = 1 Tax = *Triticum aestivum* RepID = Q1W692_WHEAT | 3 | 3 | 15 | 0.86 | 0.12 | 0.01 |
| UniRef100_G5DFC5 | 14_3_3 protein *n* = 2 Tax = *Triticum aestivum* RepID = G5DFC5_WHEAT | 7 | 11 | 107 | 0.86 | 0.06 | 0.00 |
| UniRef100_P93616 | Poly(A)_binding protein *n* = 1 Tax = *Triticum aestivum* RepID = P93616_WHEAT | 4 | 4 | 19 | 0.87 | 0.10 | 0.00 |
| UniRef100_Q08G36 | 14_3_3 protein *n* = 1 Tax = *Triticum aestivum* RepID = Q08G36_WHEAT | 8 | 12 | 94 | 0.87 | 0.04 | 0.00 |
| RFL_Contig3269 | _pep__2:77_985 | 6 | 10 | 70 | 0.87 | 0.05 | 0.00 |
| UniRef100_B2B9T8 | V_type proton ATPase subunit F *n* = 2 Tax = Triticeae RepID = B2B9T8_WHEAT | 4 | 5 | 22 | 0.87 | 0.12 | 0.01 |
| RFL_Contig1864 | _pep__3:60_566 | 2 | 3 | 14 | 0.87 | 0.10 | 0.00 |
| gi_257719940_emb_CBD33082.1_ | unnamed protein product *(Triticum aestivum)* | 3 | 4 | 15 | 0.87 | 0.09 | 0.02 |
| UniRef100_Q9FXQ9 | TaWIN1 *n* = 1 Tax = *Triticum aestivum* RepID = Q9FXQ9_WHEAT | 7 | 9 | 62 | 0.87 | 0.04 | 0.00 |
| UniRef100_F2VQK3 | Atp4_1 *n* = 1 Tax = *Triticum aestivum* RepID = F2VQK3_WHEAT | 3 | 4 | 22 | 0.87 | 0.11 | 0.01 |
| RFL_Contig1443 | _pep__3:513_887 | 2 | 2 | 8 | 0.87 | 0.15 | 0.01 |
| UniRef100_A1YE31 | Ribosomal protein L3_A3 *n* = 1 Tax = *Triticum aestivum* RepID = A1YE31_WHEAT | 8 | 13 | 67 | 0.87 | 0.04 | 0.02 |
| RFL_Contig876 | _pep__3:159_1526 | 2 | 2 | 5 | 0.87 | 0.11 | 0.02 |
| RFL_Contig3677 | _pep__3:54_725 | 2 | 3 | 6 | 0.87 | 0.10 | 0.00 |
| UniRef100_B2ZGK6 | Plastid acetyl_CoA carboxylase *n* = 50 Tax = Pooideae RepID = B2ZGK6_TRIUA | 9 | 9 | 20 | 0.87 | 0.06 | 0.00 |

**Table S2.** *Cont.*

| PROTEIN ID | DESCRIPTION | Matched Peptides | FRAMES | HITS | Ratio | SD | *p*-Value |
|---|---|---|---|---|---|---|---|
| RFL_Contig3128 | _pep__3:87_524 | 2 | 2 | 16 | 0.87 | 0.08 | 0.00 |
| UniRef100_A4K4Y7 | Alpha tubulin_4D *n* = 1 Tax = *Triticum aestivum* RepID = A4K4Y7_WHEAT | 10 | 15 | 145 | 0.88 | 0.03 | 0.00 |
| gi_219736721_emb_CAW66961.1_ | unnamed protein product (*Triticum aestivum*) | 2 | 3 | 11 | 0.88 | 0.08 | 0.01 |
| RFL_Contig773 | _pep__2:83_862 | 3 | 4 | 17 | 0.88 | 0.08 | 0.00 |
| UniRef100_A7J2I0 | Plasma membrane intrinsic protein *n* = 1 Tax = *Triticum aestivum* RepID = A7J2I0_WHEAT | 3 | 3 | 23 | 0.88 | 0.04 | 0.00 |
| RFL_Contig3347 | _pep__1:43_1236 | 2 | 2 | 14 | 0.88 | 0.12 | 0.01 |
| RFL_Contig5794 | _pep__2:89_2389 | 17 | 31 | 234 | 0.88 | 0.03 | 0.00 |
| RFL_Contig5814 | _pep__3:78_2375 | 19 | 35 | 299 | 0.88 | 0.03 | 0.00 |
| RFL_Contig3640 | _pep__1:91_3054 | 4 | 5 | 19 | 0.89 | 0.06 | 0.01 |
| gi_295422633_emb_CBL75153.1_ | unnamed protein product (*Triticum aestivum*) | 3 | 3 | 19 | 0.89 | 0.08 | 0.00 |
| gi_257672159_emb_CBD32171.1_ | unnamed protein product (*Triticum aestivum*) | 2 | 8 | 60 | 0.89 | 0.07 | 0.03 |
| gi_257672167_emb_CBD32175.1_ | unnamed protein product (*Triticum aestivum*) | 2 | 8 | 66 | 0.89 | 0.07 | 0.03 |
| UniRef100_O82073 | Sucrose synthase type I *n* = 2 Tax = *Triticum aestivum* RepID = O82073_WHEAT | 19 | 26 | 192 | 0.90 | 0.05 | 0.01 |
| gi_227473233_emb_CAY33014.1_ | unnamed protein product (*Triticum aestivum*) | 7 | 7 | 14 | 0.90 | 0.05 | 0.00 |
| gi_227295766_emb_CAY03053.1_ | unnamed protein product (*Triticum aestivum*) | 4 | 4 | 25 | 0.90 | 0.10 | 0.00 |
| gi_296514140_emb_CBM39929.1_ | unnamed protein product (*Triticum aestivum*) | 6 | 6 | 59 | 0.90 | 0.11 | 0.00 |
| gi_257672153_emb_CBD32168.1_ | unnamed protein product (*Triticum aestivum*) | 3 | 10 | 129 | 0.90 | 0.05 | 0.00 |
| gi_257672155_emb_CBD32169.1_ | unnamed protein product (*Triticum aestivum*) | 3 | 10 | 129 | 0.90 | 0.05 | 0.00 |
| RFL_Contig4300 | _pep__2:125_586 | 2 | 2 | 13 | 0.90 | 0.10 | 0.00 |
| RFL_Contig3604 | _pep__2:149_973 | 4 | 4 | 24 | 0.90 | 0.05 | 0.00 |
| RFL_Contig3076 | _pep__1:130_1965 | 4 | 4 | 15 | 0.90 | 0.06 | 0.04 |
| RFL_Contig6144 | _pep__1:88_1224 | 3 | 5 | 18 | 0.90 | 0.03 | 0.00 |
| RFL_Contig3937 | _pep__3:258_1649 | 3 | 3 | 10 | 0.90 | 0.07 | 0.01 |
| UniRef100_Q1XH04 | Beta_glucosidase *n* = 1 Tax = *Triticum aestivum* RepID = Q1XH04_WHEAT | 8 | 11 | 22 | 0.90 | 0.05 | 0.00 |
| gi_219772365_emb_CAW48568.1_ | unnamed protein product (*Triticum aestivum*) | 13 | 18 | 129 | 0.90 | 0.05 | 0.00 |
| UniRef100_P02276 | Histone H2A.2.1 *n* = 1 Tax = *Triticum aestivum* RepID = H2A2_WHEAT | 2 | 10 | 132 | 0.90 | 0.07 | 0.00 |
| UniRef100_D5MTF8 | Beta_glucosidase *n* = 1 Tax = *Triticum aestivum* RepID = D5MTF8_WHEAT | 12 | 17 | 128 | 0.91 | 0.05 | 0.00 |

**Table S2.** *Cont.*

| PROTEIN ID | DESCRIPTION | Matched Peptides | FRAMES | HITS | Ratio | SD | *p*-Value |
|---|---|---|---|---|---|---|---|
| gi_257672171_emb_CBD32177.1_ | unnamed protein product (*Triticum aestivum*) | 2 | 4 | 12 | 0.91 | 0.07 | 0.00 |
| UniRef100_Q1XIR9 | Beta_glucosidase *n* = 1 Tax = *Triticum aestivum* RepID = Q1XIR9_WHEAT | 10 | 14 | 105 | 0.91 | 0.05 | 0.00 |
| UniRef100_Q1XH05 | Beta_glucosidase *n* = 1 Tax = *Triticum aestivum* RepID = Q1XH05_WHEAT | 9 | 14 | 89 | 0.91 | 0.05 | 0.00 |
| UniRef100_P38076 | Cysteine synthase *n* = 1 Tax = *Triticum aestivum* RepID = CYSK_WHEAT | 7 | 10 | 77 | 0.91 | 0.08 | 0.00 |
| RFL_Contig1936 | _pep__1:130_579 | 2 | 2 | 18 | 0.91 | 0.07 | 0.00 |
| RFL_Contig3987 | _pep__2:107_1024 | 4 | 6 | 23 | 0.91 | 0.07 | 0.00 |
| gi_295422149_emb_CBL74911.1_ | unnamed protein product (*Triticum aestivum*) | 5 | 5 | 41 | 0.91 | 0.08 | 0.00 |
| UniRef100_P02275 | Histone H2A.1 *n* = 1 Tax = *Triticum aestivum* RepID = H2A1_WHEAT | 2 | 10 | 135 | 0.91 | 0.07 | 0.00 |
| UniRef100_F2E390 | Ribosomal protein *n* = 2 Tax = Triticeae RepID = F2E390_HORVD | 5 | 8 | 16 | 0.91 | 0.07 | 0.05 |
| RFL_Contig3661 | _pep__1:106_3954 | 2 | 2 | 15 | 0.91 | 0.15 | 0.01 |
| UniRef100_F2VQM4 | Nad9 *n* = 1 Tax=*Triticum aestivum* RepID=F2VQM4_WHEAT | 3 | 3 | 17 | 0.91 | 0.06 | 0.01 |
| UniRef100_Q53UC8 | Delta1_pyrroline_5_carboxylate synthetase *n* = 1 Tax = *Triticum aestivum* RepID = Q53UC8_WHEAT | 7 | 7 | 18 | 0.91 | 0.03 | 0.00 |
| UniRef100_Q9FVJ9 | COP alpha homolog (Fragment) *n* = 1 Tax = *Triticum aestivum* RepID = Q9FVJ9_WHEAT | 6 | 6 | 16 | 0.91 | 0.05 | 0.00 |
| RFL_Contig2923 | _pep__1:160_1344 | 6 | 7 | 26 | 0.91 | 0.05 | 0.00 |
| UniRef100_P02277 | Histone H2A.2.2 *n* = 1 Tax = *Triticum aestivum* RepID = H2A3_WHEAT | 2 | 11 | 146 | 0.91 | 0.07 | 0.00 |
| gi_257672157_emb_CBD32170.1_ | unnamed protein product (*Triticum aestivum*) | 2 | 11 | 146 | 0.91 | 0.07 | 0.00 |
| UniRef100_Q9ZRB0 | Tubulin beta_3 chain *n* = 1 Tax = *Triticum aestivum* RepID = TBB3_WHEAT | 13 | 19 | 178 | 0.91 | 0.03 | 0.00 |
| RFL_Contig2971 | _pep__1:64_600 | 5 | 5 | 30 | 0.92 | 0.04 | 0.00 |
| UniRef100_Q0PMD7 | J_domain protein *n* = 1 Tax = *Triticum aestivum* RepID = Q0PMD7_WHEAT | 2 | 2 | 7 | 0.92 | 0.08 | 0.03 |
| UniRef100_Q9SP56 | Glutathione S_transferase *n* = 1 Tax = *Triticum aestivum* RepID = Q9SP56_WHEAT | 5 | 7 | 55 | 0.92 | 0.05 | 0.00 |
| gi_257659932_emb_CBD33996.1_ | unnamed protein product (*Triticum aestivum*) | 5 | 7 | 55 | 0.92 | 0.05 | 0.00 |
| UniRef100_Q8GTB7 | Glutathione transferase F1 *n* = 1 Tax = *Triticum aestivum* RepID = Q8GTB7_WHEAT | 2 | 4 | 10 | 0.92 | 0.06 | 0.00 |
| gi_257711213_emb_CBD35086.1_ | unnamed protein product (*Triticum aestivum*) | 2 | 2 | 8 | 0.92 | 0.09 | 0.01 |
| UniRef100_Q58QF6 | Delta 1_pyrroline_5_carboxylate synthetase *n* = 1 Tax = *Triticum aestivum* RepID = Q58QF6_WHEAT | 8 | 8 | 16 | 0.92 | 0.03 | 0.00 |
| UniRef100_A4K4Z3 | Alpha tubulin_1A *n* = 1 Tax = *Triticum aestivum* RepID = A4K4Z3_WHEAT | 9 | 15 | 136 | 0.92 | 0.04 | 0.00 |
| RFL_Contig5566 | _pep__3:78_905 | 6 | 8 | 29 | 0.92 | 0.05 | 0.01 |
| RFL_Contig3435 | _pep__2:95_865 | 8 | 12 | 106 | 0.92 | 0.07 | 0.00 |

**Table S2.** *Cont.*

| PROTEIN ID | DESCRIPTION | Matched Peptides | FRAMES | HITS | Ratio | SD | *p*-Value |
|---|---|---|---|---|---|---|---|
| UniRef100_O82072 | Phosphoenolpyruvate carboxylase *n* = 1 Tax = *Triticum aestivum* RepID = O82072_WHEAT | 25 | 33 | 268 | 0.92 | 0.02 | 0.00 |
| RFL_Contig3489 | _pep__3:129_1109 | 5 | 6 | 49 | 0.92 | 0.08 | 0.00 |
| gi_295415556_emb_CBL76033.1_ | unnamed protein product (*Triticum aestivum*) | 8 | 10 | 49 | 0.93 | 0.08 | 0.00 |
| UniRef100_Q9ZRA8 | Tubulin beta_5 chain *n* = 4 Tax = Pooideae RepID = TBB5_WHEAT | 11 | 14 | 171 | 0.93 | 0.03 | 0.00 |
| UniRef100_A4K4Y1 | Alpha tubulin_2A *n* = 1 Tax = *Triticum aestivum* RepID = A4K4Y1_WHEAT | 11 | 19 | 173 | 0.93 | 0.03 | 0.00 |
| UniRef100_P83970 | Plasma membrane ATPase *n* = 1 Tax = *Triticum aestivum* RepID = PMA1_WHEAT | 20 | 28 | 190 | 0.93 | 0.05 | 0.00 |
| gi_218409192_emb_CAV27654.1_ | unnamed protein product (*Triticum aestivum*) | 20 | 24 | 197 | 0.93 | 0.03 | 0.01 |
| RFL_Contig3549 | _pep__1:88_426 | 4 | 4 | 52 | 0.93 | 0.13 | 0.00 |
| RFL_Contig3111 | _pep__2:80_682 | 2 | 3 | 36 | 0.93 | 0.07 | 0.00 |
| UniRef100_Q9ZRR5 | Tubulin alpha_3 chain *n* = 3 Tax = Triticeae RepID = TBA3_HORVU | 11 | 18 | 174 | 0.93 | 0.03 | 0.00 |
| RFL_Contig618 | _pep__2:116_700 | 2 | 2 | 3 | 0.93 | 0.09 | 0.00 |
| RFL_Contig63 | _pep__2:113_733 | 5 | 5 | 40 | 0.93 | 0.07 | 0.00 |
| RFL_Contig1587 | _pep__2:131_1375 | 11 | 11 | 62 | 0.93 | 0.04 | 0.00 |
| gi_295415558_emb_CBL76034.1_ | unnamed protein product (*Triticum aestivum*) | 7 | 11 | 85 | 0.93 | 0.08 | 0.00 |
| RFL_Contig4735 | _pep__3:96_3362 | 3 | 3 | 14 | 0.93 | 0.07 | 0.00 |
| UniRef100_Q9ZRB1 | Tubulin beta_2 chain *n* = 1 Tax = *Triticum aestivum* RepID = TBB2_WHEAT | 11 | 17 | 190 | 0.93 | 0.03 | 0.00 |
| RFL_Contig2042 | _pep__3:180_1829 | 9 | 9 | 46 | 0.94 | 0.04 | 0.00 |
| gi_257726669_emb_CBD21804.1_ | unnamed protein product (*Triticum aestivum*) | 10 | 12 | 41 | 0.94 | 0.04 | 0.00 |
| RFL_Contig4145 | _pep__2:26_508 | 3 | 3 | 17 | 0.94 | 0.09 | 0.05 |
| UniRef100_Q41583 | Initiation factor (Iso)4f p82 subunit *n* = 1 Tax = *Triticum aestivum* RepID = Q41583_WHEAT | 11 | 14 | 39 | 0.94 | 0.03 | 0.00 |
| RFL_Contig3507 | _pep__2:59_1102 | 6 | 9 | 69 | 0.94 | 0.04 | 0.00 |
| UniRef100_Q8VX48 | Phosphoglucomutase (Fragment) *n* = 1 Tax = *Triticum aestivum* RepID = Q8VX48_WHEAT | 12 | 12 | 87 | 0.94 | 0.06 | 0.00 |
| UniRef100_Q93YY0 | 68 kDa protein HP68 *n* = 1 Tax = *Triticum aestivum* RepID = Q93YY0_WHEAT | 4 | 4 | 10 | 0.94 | 0.09 | 0.00 |
| UniRef100_A5YVV3 | Glyceraldehyde_3_phosphate dehydrogenase *n* = 1 Tax = *Triticum aestivum* RepID = A5YVV3_WHEAT | 11 | 22 | 184 | 0.94 | 0.05 | 0.00 |
| UniRef100_Q9FS79 | Triosephosphate isomerase *n* = 1 Tax =*Triticum aestivum* RepID = Q9FS79_WHEAT | 9 | 12 | 74 | 0.94 | 0.06 | 0.00 |
| UniRef100_A7J2I1 | Plasma membrane intrinsic protein *n* = 1 Tax = *Triticum aestivum* RepID = A7J2I1_WHEAT | 4 | 5 | 33 | 0.94 | 0.04 | 0.00 |
| UniRef100_Q03387 | Eukaryotic initiation factor iso_4F subunit p82_34 *n* = 1 Tax = *Triticum aestivum* RepID = IF41_WHEAT | 13 | 16 | 66 | 0.94 | 0.03 | 0.00 |

**Table S2.** *Cont.*

| PROTEIN ID | DESCRIPTION | Matched Peptides | FRAMES | HITS | Ratio | SD | *p*-Value |
|---|---|---|---|---|---|---|---|
| UniRef100_B0LXM0 | S_adenosylmethionine synthase *n* = 1 Tax = *Triticum aestivum* RepID = METK_WHEAT | 5 | 9 | 67 | 0.94 | 0.05 | 0.03 |
| UniRef100_Q7X9L6 | 40S ribosomal protein (Fragment) *n* = 1 Tax = *Triticum aestivum* RepID = Q7X9L6_WHEAT | 4 | 4 | 7 | 0.94 | 0.02 | 0.00 |
| UniRef100_Q8L804 | Putative 40S ribosomal protein S3 *n* = 1 Tax = *Triticum aestivum* RepID = Q8L804_WHEAT | 6 | 7 | 53 | 0.94 | 0.02 | 0.00 |
| UniRef100_F6IB54 | Putative feruloyl transferase *n* = 1 Tax = *Triticum aestivum* RepID = F6IB54_WHEAT | 2 | 2 | 16 | 0.94 | 0.03 | 0.00 |
| RFL_Contig4437 | _pep__2:137_2578 | 6 | 6 | 24 | 0.95 | 0.10 | 0.02 |
| UniRef100_A7J2I2 | Plasma membrane intrinsic protein *n* = 1 Tax = *Triticum aestivum* RepID = A7J2I2_WHEAT | 5 | 7 | 64 | 0.95 | 0.03 | 0.00 |
| UniRef100_Q41534 | ATP synthase subunit beta *n* = 1 Tax = *Triticum aestivum* RepID = Q41534_WHEAT | 19 | 35 | 272 | 0.95 | 0.04 | 0.00 |
| UniRef100_C9EF64 | Dehydroascorbate reductase *n* = 1 Tax = *Triticum aestivum* RepID = C9EF64_WHEAT | 7 | 9 | 63 | 0.95 | 0.04 | 0.00 |
| gi_296511813_emb_CBM39048.1_ | unnamed protein product (*Triticum aestivum*) | 2 | 2 | 2 | 0.95 | 0.06 | 0.00 |
| UniRef100_Q84UH6 | Dehydroascorbate reductase *n* = 2 Tax = Triticum RepID = Q84UH6_WHEAT | 7 | 10 | 63 | 0.95 | 0.03 | 0.00 |
| RFL_Contig3778 | _pep__3:354_3464 | 4 | 4 | 18 | 0.95 | 0.05 | 0.01 |
| RFL_Contig2570 | _pep__1:94_1038 | 6 | 7 | 27 | 0.96 | 0.08 | 0.00 |
| UniRef100_Q9ZRA9 | Tubulin beta_4 chain *n* = 1 Tax = *Triticum aestivum* RepID = TBB4_WHEAT | 13 | 18 | 183 | 0.96 | 0.03 | 0.00 |
| RFL_Contig3647 | _pep__3:66_1604 | 5 | 6 | 29 | 0.96 | 0.10 | 0.01 |
| UniRef100_O82571 | Superoxide dismutase *n* = 1 Tax = *Triticum aestivum* RepID = O82571_WHEAT | 3 | 3 | 9 | 0.96 | 0.08 | 0.00 |
| RFL_Contig31 | _pep__3:72_440 | 3 | 3 | 15 | 0.96 | 0.04 | 0.05 |
| RFL_Contig1940 | _pep__1:7_954 | 6 | 8 | 40 | 0.96 | 0.05 | 0.03 |
| RFL_Contig3378 | _pep__2:140_1723 | 12 | 14 | 102 | 0.96 | 0.06 | 0.00 |
| RFL_Contig2911 | _pep__2:110_1441 | 8 | 9 | 66 | 0.96 | 0.05 | 0.00 |
| UniRef100_G4XH71 | Peptidyl_prolyl cis_trans isomerase *n* = 2 Tax = Triticeae RepID = G4XH71_9POAL | 3 | 5 | 48 | 0.96 | 0.06 | 0.00 |
| gi_257672699_emb_CBD34755.1_ | unnamed protein product (*Triticum aestivum*) | 16 | 21 | 157 | 0.97 | 0.05 | 0.00 |
| UniRef100_A7LM55 | Peptidyl_prolyl cis_trans isomerase *n* = 1 Tax = *Triticum aestivum* RepID = A7LM55_WHEAT | 3 | 7 | 63 | 0.97 | 0.06 | 0.00 |
| UniRef100_Q332R4 | ATP synthase subunit alpha *n* = 3 Tax = Triticeae RepID = Q332R4_WHEAT | 13 | 14 | 113 | 0.97 | 0.03 | 0.05 |
| UniRef100_P43650 | Putative ATP synthase protein YMF19 *n* = 2 Tax = Triticeae RepID = YMF19_WHEAT | 3 | 3 | 23 | 0.97 | 0.03 | 0.00 |
| UniRef100_C3VQ52 | Ascorbate peroxidase (Fragment) *n* = 1 Tax = *Triticum aestivum* RepID = C3VQ52_WHEAT | 9 | 11 | 76 | 0.97 | 0.07 | 0.01 |
| UniRef100_B9A8E2 | Protein disulfide isomerase *n* = 1 Tax = *Triticum aestivum* RepID = B9A8E2_WHEAT | 11 | 13 | 91 | 0.97 | 0.04 | 0.00 |
| UniRef100_B9A8E3 | Protein disulfide isomerase *n* = 1 Tax = *Triticum aestivum* RepID = B9A8E3_WHEAT | 13 | 15 | 65 | 0.97 | 0.04 | 0.00 |

**Table S2.** *Cont.*

| PROTEIN ID | DESCRIPTION | Matched Peptides | FRAMES | HITS | Ratio | SD | *p*-Value |
|---|---|---|---|---|---|---|---|
| UniRef100_B2BA44 | Vacuolar proton ATPase subunit H *n* = 1 Tax = *Triticum aestivum* RepID = B2BA44_WHEAT | 9 | 11 | 73 | 0.97 | 0.05 | 0.04 |
| UniRef100_P52589 | Protein disulfide_isomerase *n* = 1 Tax = *Triticum aestivum* RepID = PDI_WHEAT | 11 | 12 | 53 | 0.97 | 0.04 | 0.00 |
| UniRef100_Q8L806 | Putative ribosomal protein S18 *n* = 1 Tax = *Triticum aestivum* RepID = Q8L806_WHEAT | 3 | 3 | 19 | 0.97 | 0.05 | 0.01 |
| RFL_Contig3770 | _pep__3:33_1130 | 11 | 18 | 152 | 0.97 | 0.05 | 0.00 |
| UniRef100_Q9ZRA7 | Beta_tubulin 6 (Fragment) *n* = 1 Tax = *Triticum aestivum* RepID = Q9ZRA7_WHEAT | 6 | 10 | 47 | 0.97 | 0.04 | 0.00 |
| RFL_Contig3321 | _pep__3:156_1730 | 10 | 14 | 106 | 0.97 | 0.04 | 0.00 |
| RFL_Contig4151 | _pep__3:126_5297 | 29 | 34 | 190 | 0.97 | 0.03 | 0.00 |
| gi_257711217_emb_CBD35088.1_ | unnamed protein product (*Triticum aestivum*) | 2 | 2 | 26 | 0.97 | 0.07 | 0.05 |
| RFL_Contig3538 | _pep__1:112_1083 | 6 | 7 | 60 | 0.98 | 0.05 | 0.01 |
| RFL_Contig5895 | _pep__2:608_3703 | 3 | 3 | 33 | 0.98 | 0.06 | 0.00 |
| RFL_Contig4724 | _pep__3:228_3104 | 8 | 10 | 46 | 0.98 | 0.07 | 0.01 |
| RFL_Contig3118 | _pep__2:137_1474 | 3 | 3 | 36 | 0.98 | 0.08 | 0.02 |
| UniRef100_D8L9U6 | Eukaryotic translation initiation factor_putative_ expressed *n* = 1 Tax = *Triticum aestivum* RepID = D8L9U6_WHEAT | 6 | 6 | 24 | 0.98 | 0.08 | 0.03 |
| RFL_Contig5602 | _pep__2:71_748 | 6 | 9 | 79 | 0.98 | 0.04 | 0.00 |
| UniRef100_F4Y592 | Heat shock protein 90 *n* = 2 Tax = Triticum RepID = F4Y592_WHEAT | 8 | 8 | 16 | 0.98 | 0.05 | 0.00 |
| UniRef100_A3KLL4 | Malate dehydrogenase *n* = 1 Tax = *Triticum aestivum* RepID = A3KLL4_WHEAT | 6 | 10 | 99 | 0.98 | 0.08 | 0.04 |
| RFL_Contig4129 | _pep__1:262_1017 | 3 | 4 | 18 | 0.98 | 0.04 | 0.01 |
| RFL_Contig259 | _pep__3:63_1403 | 13 | 17 | 162 | 0.98 | 0.05 | 0.00 |
| UniRef100_F4Y594 | Heat shock protein 90 *n* = 2 Tax = Triticeae RepID = F4Y594_WHEAT | 8 | 10 | 14 | 0.98 | 0.05 | 0.00 |
| RFL_Contig3043 | _pep__1:235_2394 | 7 | 7 | 42 | 0.98 | 0.06 | 0.00 |
| UniRef100_Q03033 | Elongation factor 1_alpha *n* = 3 Tax = Triticeae RepID = EF1A_WHEAT | 10 | 22 | 176 | 0.99 | 0.05 | 0.00 |
| UniRef100_F4Y589 | Heat shock protein 90 *n* = 1 Tax = *Triticum aestivum* RepID = F4Y589_WHEAT | 6 | 7 | 24 | 0.99 | 0.04 | 0.00 |
| RFL_Contig3625 | _pep__3:405_2606 | 4 | 4 | 9 | 0.99 | 0.15 | 0.03 |
| RFL_Contig3308 | _pep__1:88_2154 | 10 | 11 | 82 | 0.99 | 0.04 | 0.04 |
| UniRef100_Q9SAU8 | HSP70 *n* = 1 Tax = *Triticum aestivum* RepID = Q9SAU8_WHEAT | 15 | 19 | 162 | 0.99 | 0.03 | 0.00 |
| gi_296511811_emb_CBM39047.1_ | unnamed protein product (*Triticum aestivum*) | 10 | 10 | 68 | 0.99 | 0.04 | 0.00 |

**Table S3.** The list of proteins identified in seminal roots of the wheat transgenic line and wild type under waterlogging.

| PROTEIN ID | DESCRIPTION | Matched Peptides | FRAMES | HITS | Ratio | SD | *p*-Value |
|---|---|---|---|---|---|---|---|
| Increase | | | | | | | |
| UniRef100_Q8S3J5 | Ferredoxin *n* = 1 Tax = *Triticum aestivum* RepID = Q8S3J5_WHEAT | 2 | 2 | 6 | 2.02 | 2.29 | 0.01 |
| UniRef100_F8S6T6 | Pathogenesis_related protein 1_6 *n* = 1 Tax = *Triticum aestivum* RepID = F8S6T6_WHEAT | 2 | 3 | 15 | 1.97 | 0.66 | 0.00 |
| UniRef100_Q9M7S5 | Elongation factor_2 (Fragment) *n* = 1 Tax = *Triticum aestivum* RepID = Q9M7S5_WHEAT | 2 | 2 | 9 | 1.87 | 1.15 | 0.03 |
| UniRef100_F8S6U7 | Pathogenesis_related protein 1_17 *n* = 1 Tax = *Triticum aestivum* RepID = F8S6U7_WHEAT | 5 | 8 | 82 | 1.53 | 0.10 | 0.00 |
| RFL_Contig2626 | _pep__2:92_1075 | 2 | 2 | 2 | 1.35 | 0.11 | 0.02 |
| RFL_Contig2940 | _pep__2:62_583 | 2 | 3 | 22 | 1.33 | 0.40 | 0.00 |
| UniRef100_Q7XYB6 | Elongation factor (Fragment) *n* = 1 Tax = *Triticum aestivum* RepID = Q7XYB6_WHEAT | 2 | 3 | 35 | 1.30 | 0.30 | 0.01 |
| RFL_Contig246 | _pep__3:57_590 | 2 | 2 | 3 | 1.24 | 0.16 | 0.01 |
| RFL_Contig2363 | _pep__2:65_1267 | 4 | 5 | 20 | 1.22 | 0.12 | 0.00 |
| RFL_Contig5163 | _pep__2:125_1489 | 3 | 3 | 14 | 1.22 | 0.09 | 0.00 |
| RFL_Contig2035 | _pep__1:49_1818 | 2 | 2 | 3 | 1.22 | 0.34 | 0.02 |
| UniRef100_A2WN93 | Calmodulin_1 *n* = 18 Tax = Magnoliophyta RepID = CALM1_ORYSI | 3 | 3 | 22 | 1.21 | 0.18 | 0.00 |
| UniRef100_Q84MJ5 | Methylmalonate semialdehyde dehydrogenase (Fragment) *n* = 1 Tax = *Triticum aestivum* RepID = Q84MJ5_WHEAT | 3 | 5 | 56 | 1.19 | 0.16 | 0.00 |
| RFL_Contig3223 | _pep__2:116_607 | 5 | 6 | 28 | 1.19 | 0.13 | 0.00 |
| UniRef100_A9U8G4 | Alcohol dehydrogenase ADH1A *n* = 2 Tax = Triticum RepID = A9U8G4_WHEAT | 9 | 14 | 89 | 1.18 | 0.07 | 0.00 |
| RFL_Contig3037 | _pep__1:118_786 | 3 | 3 | 26 | 1.18 | 0.11 | 0.00 |
| RFL_Contig4868 | _pep__3:54_1019 | 3 | 3 | 6 | 1.17 | 0.12 | 0.00 |
| UniRef100_I1HEK5 | Uncharacterized protein *n* = 2 Tax = Pooideae RepID = I1HEK5_BRADI | 3 | 3 | 13 | 1.16 | 0.17 | 0.00 |
| RFL_Contig2784 | _pep__2:128_631 | 3 | 4 | 40 | 1.16 | 0.13 | 0.00 |
| UniRef100_Q8GTC0 | Glutathione transferase F3 *n* = 1 Tax = *Triticum aestivum* RepID = Q8GTC0_WHEAT | 2 | 3 | 35 | 1.16 | 0.19 | 0.00 |
| UniRef100_A4GSN7 | Ferritin *n* = 1 Tax = *Triticum aestivum* RepID = A4GSN7_WHEAT | 3 | 3 | 4 | 1.14 | 0.15 | 0.00 |
| UniRef100_D1MBU0 | Type II metacaspase *n* = 1 Tax = *Triticum aestivum* RepID = D1MBU0_WHEAT | 5 | 5 | 39 | 1.14 | 0.11 | 0.00 |
| RFL_Contig3391 | _pep__3:87_767 | 2 | 2 | 16 | 1.13 | 0.06 | 0.00 |
| UniRef100_A7XDG5 | Peptidyl_prolyl cis_trans isomerase *n* = 1 Tax = *Triticum aestivum* RepID = A7XDG5_WHEAT | 2 | 2 | 15 | 1.13 | 0.08 | 0.00 |
| RFL_Contig2286 | _pep__2:107_1555 | 8 | 12 | 74 | 1.13 | 0.07 | 0.00 |

**Table S3.** *Cont.*

| PROTEIN ID | DESCRIPTION | Matched Peptides | FRAMES | HITS | Ratio | SD | *p*-Value |
|---|---|---|---|---|---|---|---|
| RFL_Contig2640 | _pep__2:101_1549 | 10 | 13 | 40 | 1.13 | 0.07 | 0.00 |
| UniRef100_A9U8G5 | Alcohol dehydrogenase ADH1D *n* = 1 Tax = *Triticum aestivum* RepID = A9U8G5_WHEAT | 9 | 15 | 80 | 1.12 | 0.08 | 0.00 |
| RFL_Contig1157 | _pep__1:55_1080 | 6 | 7 | 57 | 1.11 | 0.13 | 0.00 |
| gi_219914282_emb_CAW74910.1_ | unnamed protein product (*Triticum aestivum*) | 4 | 5 | 65 | 1.10 | 0.04 | 0.00 |
| UniRef100_B8YEL1 | Ferredoxin_nitrite reductase *n* = 1 Tax = *Triticum aestivum* RepID = B8YEL1_WHEAT | 5 | 7 | 34 | 1.10 | 0.08 | 0.04 |
| UniRef100_O04437 | Glutathione S_transferase *n* = 1 Tax = *Triticum aestivum* RepID = GSTZ_WHEAT | 2 | 2 | 10 | 1.10 | 0.16 | 0.04 |
| UniRef100_H8ZI06 | HSP70 (Fragment) *n* = 1 Tax = *Triticum aestivum* RepID = H8ZI06_WHEAT | 4 | 5 | 22 | 1.10 | 0.07 | 0.00 |
| UniRef100_A9U8G1 | Alcohol dehydrogenase ADH2D *n* = 1 Tax = *Triticum aestivum* RepID = A9U8G1_WHEAT | 6 | 9 | 43 | 1.09 | 0.10 | 0.00 |
| RFL_Contig3621 | _pep__3:84_2090 | 6 | 6 | 20 | 1.09 | 0.06 | 0.00 |
| gi_359828768_gb_AEV76986.1_ | beta_glucosidase 4_ partial (*Triticum aestivum*) | 2 | 2 | 7 | 1.08 | 0.18 | 0.00 |
| RFL_Contig3509 | _pep__2:197_1150 | 2 | 2 | 15 | 1.08 | 0.17 | 0.04 |
| RFL_Contig2792 | _pep__2:62_1270 | 2 | 2 | 14 | 1.07 | 0.14 | 0.00 |
| UniRef100_D8L9G6 | Phosphorylase *n* = 1 Tax = *Triticum aestivum* RepID = D8L9G6_WHEAT | 5 | 6 | 9 | 1.06 | 0.14 | 0.04 |
| UniRef100_Q6RUJ1 | Glutamine synthetase *n* = 1 Tax = *Triticum aestivum* RepID = Q6RUJ1_WHEAT | 5 | 6 | 20 | 1.05 | 0.16 | 0.01 |
| UniRef100_D8L9B3 | Putative PDI_like protein *n* = 1 Tax = *Triticum aestivum* RepID = D8L9B3_WHEAT | 6 | 9 | 84 | 1.05 | 0.10 | 0.00 |
| RFL_Contig4839 | _pep__3:18_1208 | 4 | 5 | 22 | 1.05 | 0.13 | 0.01 |
| RFL_Contig37 | _pep__3:117_1193 | 7 | 9 | 49 | 1.05 | 0.05 | 0.01 |
| gi_296523708_emb_CBM36708.1_ | unnamed protein product (*Triticum aestivum*) | 6 | 6 | 11 | 1.05 | 0.10 | 0.00 |
| gi_291047854_emb_CBK51440.1_ | unnamed protein product (*Triticum aestivum*) | 3 | 3 | 4 | 1.04 | 0.09 | 0.05 |
| UniRef100_O82073 | Sucrose synthase type I *n* = 2 Tax = *Triticum aestivum* RepID = O82073_WHEAT | 24 | 50 | 419 | 1.04 | 0.03 | 0.00 |
| gi_257667483_emb_CBD31968.1_ | unnamed protein product (*Triticum aestivum*) | 2 | 3 | 26 | 1.04 | 0.18 | 0.00 |
| RFL_Contig3777 | _pep__2:104_1627 | 2 | 3 | 11 | 1.04 | 0.16 | 0.00 |
| UniRef100_P20973 | Ubiquitin_activating enzyme E1 1 *n* = 1 Tax = *Triticum aestivum* RepID = UBE11_WHEAT | 10 | 12 | 71 | 1.04 | 0.07 | 0.00 |
| RFL_Contig2547 | _pep__2:83_1108 | 6 | 8 | 80 | 1.03 | 0.07 | 0.00 |
| UniRef100_Q8VWM9 | Fructose_bisphosphate aldolase (Fragment) *n* = 1 Tax = *Triticum aestivum* RepID = Q8VWM9_WHEAT | 9 | 15 | 110 | 1.03 | 0.06 | 0.05 |
| RFL_Contig3134 | _pep__3:120_1937 | 5 | 5 | 19 | 1.03 | 0.11 | 0.00 |
| UniRef100_F2CQ27 | Predicted protein *n* = 2 Tax = Triticeae RepID = F2CQ27_HORVD | 4 | 4 | 32 | 1.03 | 0.03 | 0.00 |

**Table S3.** *Cont.*

| PROTEIN ID | DESCRIPTION | Matched Peptides | FRAMES | HITS | Ratio | SD | *p*-Value |
|---|---|---|---|---|---|---|---|
| UniRef100_C1J959 | Fructose_bisphosphate aldolase *n* = 1 Tax = *Triticum aestivum* RepID = C1J959_WHEAT | 13 | 21 | 132 | 1.03 | 0.05 | 0.04 |
| RFL_Contig5913 | _pep__1:166_957 | 5 | 6 | 47 | 1.02 | 0.16 | 0.03 |
| RFL_Contig1280 | _pep__3:195_1787 | 3 | 5 | 33 | 1.02 | 0.14 | 0.00 |
| UniRef100_P41378 | Eukaryotic initiation factor 4A *n* = 1 Tax = *Triticum aestivum* RepID = IF4A_WHEAT | 7 | 8 | 15 | 1.01 | 0.05 | 0.00 |
| UniRef100_Q401N7 | Aspartic proteinase *n* = 1 Tax = *Triticum aestivum* RepID = Q401N7_WHEAT | 6 | 7 | 27 | 1.01 | 0.08 | 0.03 |
| Decrease | | | | | | | |
| RFL_Contig2907 | _pep__2:905_1681 | 4 | 4 | 14 | 0.56 | 0.06 | 0.00 |
| UniRef100_H2KXF7 | Pathogenesis_related protein *n* = 1 Tax = *Triticum aestivum* RepID = H2KXF7_WHEAT | 4 | 5 | 17 | 0.61 | 0.10 | 0.00 |
| UniRef100_H9CWE9 | 12_oxo_phytodienoic acid reductase *n* = 1 Tax = *Triticum aestivum* RepID = H9CWE9_WHEAT | 2 | 2 | 9 | 0.73 | 0.17 | 0.03 |
| UniRef100_Q8GTB9 | Glutathione transferase F4 *n* = 1 Tax = *Triticum aestivum* RepID = Q8GTB9_WHEAT | 2 | 3 | 16 | 0.76 | 0.07 | 0.00 |
| UniRef100_D8LAL9 | Hydroquinone glucosyltransferase_ putative_ expressed *n* = 1 Tax = *Triticum aestivum* RepID = D8LAL9_WHEAT | 2 | 2 | 15 | 0.77 | 0.17 | 0.02 |
| UniRef100_Q43199 | Adenine phosphoribosyltransferase 1 *n* = 1 Tax = *Triticum aestivum* RepID = APT1_WHEAT | 4 | 5 | 36 | 0.78 | 0.06 | 0.00 |
| RFL_Contig2919 | _pep__2:80_598 | 3 | 3 | 7 | 0.79 | 0.04 | 0.00 |
| UniRef100_P38076 | Cysteine synthase *n* = 1 Tax = *Triticum aestivum* RepID = CYSK_WHEAT | 8 | 9 | 90 | 0.79 | 0.06 | 0.00 |
| UniRef100_P59232 | Ubiquitin_40S ribosomal protein S27a_2 *n* = 18 Tax = Eukaryota RepID = R27AB_ARATH | 4 | 8 | 63 | 0.79 | 0.08 | 0.00 |
| UniRef100_A1YE34 | Ribosomal protein L3_B2 *n* = 1 Tax = *Triticum aestivum* RepID = A1YE34_WHEAT | 6 | 9 | 23 | 0.79 | 0.05 | 0.00 |
| RFL_Contig2772 | _pep__3:60_4160 | 6 | 6 | 18 | 0.80 | 0.10 | 0.01 |
| UniRef100_A1YE31 | Ribosomal protein L3_A3 *n* = 1 Tax = *Triticum aestivum* RepID = A1YE31_WHEAT | 8 | 12 | 72 | 0.80 | 0.05 | 0.00 |
| RFL_Contig3907 | _pep__3:174_1214 | 2 | 2 | 11 | 0.80 | 0.11 | 0.00 |
| UniRef100_B5B3P8 | Pathogenesis related protein 10 *n* = 2 Tax = commelinids RepID = B5B3P8_WHEAT | 3 | 3 | 13 | 0.80 | 0.12 | 0.00 |
| UniRef100_P04464 | Calmodulin *n* = 1 Tax = *Triticum aestivum* RepID = CALM_WHEAT | 4 | 4 | 17 | 0.80 | 0.06 | 0.00 |
| UniRef100_P43650 | Putative ATP synthase protein YMF19 *n* = 2 Tax = Triticeae RepID = YMF19_WHEAT | 3 | 3 | 19 | 0.80 | 0.06 | 0.00 |
| UniRef100_Q8RW03 | Glutathione transferase *n* = 1 Tax = *Triticum aestivum* RepID = Q8RW03_WHEAT | 5 | 5 | 17 | 0.81 | 0.07 | 0.00 |
| UniRef100_Q08G39 | Ribosomal protein L3 *n* = 1 Tax = *Triticum aestivum* RepID = Q08G39_WHEAT | 8 | 11 | 53 | 0.81 | 0.05 | 0.00 |
| UniRef100_Q8LL15 | Glutathione_S_transferase 28e45 *n* = 1 Tax = *Triticum aestivum* RepID = Q8LL15_WHEAT | 3 | 3 | 31 | 0.81 | 0.13 | 0.00 |
| gi_295422149_emb_CBL74911.1_ | unnamed protein product (*Triticum aestivum*) | 6 | 7 | 39 | 0.81 | 0.06 | 0.00 |

**Table S3.** *Cont.*

| PROTEIN ID | DESCRIPTION | Matched Peptides | FRAMES | HITS | Ratio | SD | *p*-Value |
|---|---|---|---|---|---|---|---|
| RFL_Contig1200 | _pep__1:70_1410 | 2 | 2 | 3 | 0.81 | 0.10 | 0.00 |
| UniRef100_Q5I7L5 | 60S ribosomal protein L36 *n* = 1 Tax = *Triticum aestivum* RepID = Q5I7L5_WHEAT | 2 | 2 | 17 | 0.82 | 0.14 | 0.00 |
| UniRef100_A4K4Y7 | Alpha tubulin_4D *n* = 1 Tax = *Triticum aestivum* RepID = A4K4Y7_WHEAT | 12 | 17 | 162 | 0.82 | 0.06 | 0.00 |
| UniRef100_Q7XYE4 | 40S ribosomal protein S9 (Fragment) *n* = 1 Tax = *Triticum aestivum* RepID = Q7XYE4_WHEAT | 2 | 3 | 26 | 0.82 | 0.02 | 0.00 |
| UniRef100_Q9ZRA8 | Tubulin beta_5 chain *n* = 4 Tax = Pooideae RepID = TBB5_WHEAT | 13 | 18 | 183 | 0.82 | 0.05 | 0.00 |
| RFL_Contig2002 | _pep__2:95_1294 | 2 | 2 | 3 | 0.83 | 0.18 | 0.05 |
| gi_295415558_emb_CBL76034.1_ | unnamed protein product (*Triticum aestivum*) | 8 | 10 | 97 | 0.83 | 0.06 | 0.00 |
| UniRef100_Q8RW02 | Glutathione transferase *n* = 1 Tax = *Triticum aestivum* RepID = Q8RW02_WHEAT | 4 | 5 | 38 | 0.83 | 0.07 | 0.00 |
| gi_218389472_emb_CAV25023.1_ | unnamed protein product (*Triticum aestivum*) | 2 | 2 | 9 | 0.83 | 0.08 | 0.00 |
| gi_295422633_emb_CBL75153.1_ | unnamed protein product (*Triticum aestivum*) | 5 | 5 | 8 | 0.83 | 0.06 | 0.00 |
| gi_295415556_emb_CBL76033.1_ | unnamed protein product (*Triticum aestivum*) | 8 | 10 | 74 | 0.83 | 0.07 | 0.00 |
| UniRef100_Q5I5K7 | Small GTP_binding protein *n* = 1 Tax = *Triticum aestivum* RepID = Q5I5K7_WHEAT | 7 | 8 | 38 | 0.83 | 0.07 | 0.00 |
| RFL_Contig3884 | _pep__1:124_2052 | 3 | 3 | 9 | 0.84 | 0.09 | 0.01 |
| UniRef100_I0JTW5 | Eukaryotic translation initiation factor 3 subunit A_ putative_ expressed *n* = 1 Tax = *Triticum aestivum* RepID = I0JTW5_WHEAT | 2 | 2 | 3 | 0.84 | 0.14 | 0.00 |
| RFL_Contig2682 | _pep__2:98_1111 | 13 | 17 | 138 | 0.84 | 0.04 | 0.00 |
| UniRef100_A4K4Y1 | Alpha tubulin_2A *n* = 1 Tax = *Triticum aestivum* RepID = A4K4Y1_WHEAT | 12 | 19 | 192 | 0.84 | 0.06 | 0.00 |
| UniRef100_Q9ZRR5 | Tubulin alpha_3 chain *n* = 3 Tax = Triticeae RepID = TBA3_HORVU | 12 | 18 | 191 | 0.84 | 0.06 | 0.00 |
| gi_227295766_emb_CAY03053.1_ | unnamed protein product (*Triticum aestivum*) | 3 | 4 | 15 | 0.84 | 0.07 | 0.00 |
| UniRef100_Q332R4 | ATP synthase subunit alpha *n* = 3 Tax = Triticeae RepID = Q332R4_WHEAT | 13 | 16 | 127 | 0.85 | 0.04 | 0.00 |
| RFL_Contig501 | _pep__1:91_1434 | 12 | 18 | 111 | 0.85 | 0.05 | 0.01 |
| UniRef100_A4K4Z0 | Alpha tubulin_5B *n* = 1 Tax = *Triticum aestivum* RepID = A4K4Z0_WHEAT | 4 | 7 | 72 | 0.85 | 0.07 | 0.00 |
| RFL_Contig2895 | _pep__1:73_1257 | 6 | 6 | 40 | 0.85 | 0.05 | 0.00 |
| UniRef100_A0MA43 | Ran_binding protein *n* = 1 Tax = *Triticum aestivum* RepID = A0MA43_WHEAT | 2 | 2 | 12 | 0.85 | 0.17 | 0.05 |
| RFL_Contig3526 | _pep__1:115_1941 | 9 | 11 | 92 | 0.85 | 0.06 | 0.00 |
| UniRef100_A4K4Z3 | Alpha tubulin_1A *n* = 1 Tax = *Triticum aestivum* RepID = A4K4Z3_WHEAT | 10 | 16 | 161 | 0.86 | 0.07 | 0.00 |
| UniRef100_B4F6F0 | Root peroxidase *n* = 1 Tax = *Triticum aestivum* RepID = B4F6F0_WHEAT | 9 | 12 | 75 | 0.86 | 0.08 | 0.00 |

**Table S3.** *Cont.*

| PROTEIN ID | DESCRIPTION | Matched Peptides | FRAMES | HITS | Ratio | SD | *p*-Value |
|---|---|---|---|---|---|---|---|
| UniRef100_Q8RW00 | Glutathione transferase *n* = 1 Tax = *Triticum aestivum* RepID = Q8RW00_WHEAT | 4 | 5 | 32 | 0.86 | 0.09 | 0.00 |
| RFL_Contig2924 | _pep__1:124_831 | 3 | 3 | 21 | 0.86 | 0.09 | 0.00 |
| UniRef100_Q9ZRA7 | Beta_tubulin 6 (Fragment) *n* = 1 Tax = *Triticum aestivum* RepID = Q9ZRA7_WHEAT | 8 | 13 | 79 | 0.86 | 0.06 | 0.00 |
| UniRef100_B4F6E7 | Root peroxidase *n* = 1 Tax = *Triticum aestivum* RepID = B4F6E7_WHEAT | 12 | 17 | 156 | 0.86 | 0.06 | 0.00 |
| gi_226885722_emb_CAX86813.1_ | unnamed protein product (*Triticum aestivum*) | 2 | 2 | 16 | 0.86 | 0.07 | 0.00 |
| UniRef100_Q9ZRA9 | Tubulin beta_4 chain *n* = 1 Tax = *Triticum aestivum* RepID= TBB4_WHEAT | 13 | 20 | 202 | 0.86 | 0.05 | 0.00 |
| UniRef100_B4F6E6 | Root peroxidase *n* = 1 Tax = *Triticum aestivum* RepID = B4F6E6_WHEAT | 12 | 17 | 158 | 0.86 | 0.07 | 0.00 |
| UniRef100_Q9ZRB0 | Tubulin beta_3 chain *n* = 1 Tax = *Triticum aestivum* RepID = TBB3_WHEAT | 13 | 21 | 190 | 0.86 | 0.05 | 0.00 |
| gi_259662493_emb_CBG02800.1_ | unnamed protein product (*Triticum aestivum*) | 4 | 4 | 21 | 0.86 | 0.06 | 0.00 |
| gi_259662491_emb_CBG02799.1_ | unnamed protein product (*Triticum aestivum*) | 4 | 4 | 11 | 0.86 | 0.06 | 0.00 |
| RFL_Contig478 | _pep__1:70_783 | 4 | 4 | 26 | 0.87 | 0.06 | 0.00 |
| RFL_Contig1627 | _pep__1:127_1104 | 3 | 3 | 12 | 0.87 | 0.10 | 0.00 |
| RFL_Contig3205 | _pep__1:145_2199 | 3 | 4 | 7 | 0.87 | 0.04 | 0.00 |
| RFL_Contig259 | _pep__3:63_1403 | 17 | 24 | 207 | 0.87 | 0.03 | 0.00 |
| UniRef100_B4F6E5 | Root peroxidase *n* = 1 Tax = *Triticum aestivum* RepID = B4F6E5_WHEAT | 10 | 14 | 107 | 0.87 | 0.07 | 0.00 |
| UniRef100_C6ETB5 | Class III peroxidase *n* = 1 Tax = *Triticum aestivum* RepID = C6ETB5_WHEAT | 2 | 4 | 16 | 0.87 | 0.05 | 0.01 |
| RFL_Contig2129 | _pep__1:19_1374 | 2 | 3 | 21 | 0.87 | 0.10 | 0.00 |
| RFL_Contig2512 | _pep__3:63_794 | 3 | 3 | 14 | 0.88 | 0.07 | 0.04 |
| UniRef100_Q0PMD7 | J_domain protein *n* = 1 Tax = *Triticum aestivum* RepID = Q0PMD7_WHEAT | 3 | 3 | 7 | 0.88 | 0.05 | 0.00 |
| RFL_Contig2839 | _pep__3:132_584 | 2 | 3 | 8 | 0.88 | 0.14 | 0.00 |
| RFL_Contig5814 | _pep__3:78_2375 | 20 | 33 | 311 | 0.88 | 0.05 | 0.00 |
| UniRef100_B4F6F2 | Root peroxidase *n* = 1 Tax = *Triticum aestivum* RepID = B4F6F2_WHEAT | 10 | 13 | 135 | 0.88 | 0.07 | 0.00 |
| RFL_Contig2680 | _pep__1:142_753 | 3 | 3 | 4 | 0.88 | 0.06 | 0.00 |
| UniRef100_A7J2I2 | Plasma membrane intrinsic protein *n* = 1 Tax = *Triticum aestivum* RepID = A7J2I2_WHEAT | 6 | 8 | 55 | 0.88 | 0.05 | 0.00 |
| UniRef100_F4Y589 | Heat shock protein 90 *n* = 1 Tax = *Triticum aestivum* RepID = F4Y589_WHEAT | 4 | 5 | 12 | 0.89 | 0.07 | 0.00 |
| UniRef100_P51823 | ADP_ribosylation factor 2 *n* = 6 Tax = Poaceae RepID = ARF2_ORYSJ | 4 | 6 | 66 | 0.89 | 0.07 | 0.00 |
| RFL_Contig4822 | _pep__3:99_1934 | 2 | 2 | 2 | 0.89 | 0.21 | 0.05 |

**Table S3.** *Cont.*

| PROTEIN ID | DESCRIPTION | Matched Peptides | FRAMES | HITS | Ratio | SD | *p*-Value |
|---|---|---|---|---|---|---|---|
| RFL_Contig1072 | _pep__1:223_2271 | 6 | 6 | 24 | 0.89 | 0.08 | 0.01 |
| UniRef100_A7J2I1 | Plasma membrane intrinsic protein *n* = 1 Tax = *Triticum aestivum* RepID = A7J2I1_WHEAT | 5 | 7 | 32 | 0.89 | 0.05 | 0.00 |
| UniRef100_D8L9U6 | Eukaryotic translation initiation factor_putative_ expressed *n* = 1 Tax = *Triticum aestivum* RepID = D8L9U6_WHEAT | 5 | 6 | 22 | 0.89 | 0.09 | 0.00 |
| UniRef100_C6ETB6 | Class III peroxidase *n* = 1 Tax = *Triticum aestivum* RepID = C6ETB6_WHEAT | 3 | 6 | 23 | 0.89 | 0.05 | 0.01 |
| RFL_Contig3253 | _pep__1:121_996 | 6 | 7 | 50 | 0.89 | 0.04 | 0.00 |
| gi_330728034_emb_CCA64660.1_ | unnamed protein product (*Triticum aestivum*) | 4 | 5 | 41 | 0.89 | 0.10 | 0.02 |
| UniRef100_Q6IY71 | Mitochondrial ATP synthase *n* = 1 Tax = *Triticum aestivum* RepID = Q6IY71_WHEAT | 5 | 5 | 28 | 0.89 | 0.09 | 0.01 |
| RFL_Contig2992 | _pep__3:111_908 | 2 | 2 | 3 | 0.89 | 0.06 | 0.02 |
| RFL_Contig4724 | _pep__3:228_3104 | 9 | 11 | 39 | 0.90 | 0.05 | 0.01 |
| RFL_Contig3855 | _pep__3:126_1601 | 9 | 9 | 42 | 0.90 | 0.04 | 0.00 |
| RFL_Contig1865 | _pep__2:104_901 | 8 | 10 | 59 | 0.90 | 0.05 | 0.01 |
| UniRef100_F4Y592 | Heat shock protein 90 *n* = 2 Tax = Triticum RepID = F4Y592_WHEAT | 9 | 13 | 39 | 0.90 | 0.04 | 0.00 |
| gi_227248192_emb_CAY07658.1_ | unnamed protein product (*Triticum aestivum*) | 6 | 8 | 61 | 0.90 | 0.06 | 0.00 |
| RFL_Contig5048 | _pep__2:134_1606 | 2 | 2 | 2 | 0.90 | 0.03 | 0.01 |
| RFL_Contig3532 | _pep__3:99_1424 | 8 | 8 | 9 | 0.90 | 0.03 | 0.03 |
| UniRef100_Q9LRJ0 | Glucose_6_phosphate 1_dehydrogenase *n* = 1 Tax = *Triticum aestivum* RepID = Q9LRJ0_WHEAT | 10 | 11 | 51 | 0.90 | 0.07 | 0.00 |
| RFL_Contig3581 | _pep__2:92_883 | 3 | 3 | 16 | 0.90 | 0.09 | 0.01 |
| RFL_Contig1629 | _pep__2:140_1102 | 7 | 11 | 66 | 0.90 | 0.04 | 0.00 |
| gi_313103631_pdb_3IZ6_D | Chain D_ Localization Of The Small Subunit Ribosomal Proteins Into A 5.5 A Cryo_Em Map of *Triticum aestivum* Translating 80s Ribosome | 4 | 4 | 27 | 0.90 | 0.07 | 0.01 |
| gi_313103629_pdb_3IZ6_A | Chain A_ Localization Of The Small Subunit Ribosomal Proteins Into A 5.5 A Cryo_Em Map of *Triticum aestivum* Translating 80s Ribosome | 6 | 6 | 31 | 0.91 | 0.04 | 0.00 |
| RFL_Contig5445 | _pep__1:130_1044 | 3 | 3 | 22 | 0.91 | 0.06 | 0.00 |
| UniRef100_F4Y5B2 | Heat shock protein 90 *n* = 5 Tax = Triticeae RepID = F4Y5B2_AEGTA | 13 | 19 | 119 | 0.91 | 0.04 | 0.00 |
| UniRef100_Q03387 | Eukaryotic initiation factor iso_4F subunit p82_34 *n* = 1 Tax = *Triticum aestivum* RepID = IF41_WHEAT | 13 | 15 | 71 | 0.91 | 0.05 | 0.00 |
| gi_257672781_emb_CBD34794.1_ | unnamed protein product (*Triticum aestivum*) | 3 | 3 | 25 | 0.91 | 0.09 | 0.00 |

**Table S3.** *Cont.*

| PROTEIN ID | DESCRIPTION | Matched Peptides | FRAMES | HITS | Ratio | SD | *p*-Value |
|---|---|---|---|---|---|---|---|
| RFL_Contig3279 | _pep__2:122_1471 | 5 | 6 | 32 | 0.91 | 0.06 | 0.00 |
| UniRef100_Q41591 | Voltage dependent anion channel (VDAC) *n* = 1 Tax = *Triticum aestivum* RepID = Q41591_WHEAT | 5 | 5 | 21 | 0.91 | 0.05 | 0.02 |
| RFL_Contig5794 | _pep__2:89_2389 | 16 | 26 | 238 | 0.91 | 0.05 | 0.00 |
| UniRef100_Q9M7C2 | Plasma membrane intrinsic protein 3 *n* = 1 Tax = *Triticum aestivum* RepID = Q9M7C2_WHEAT | 3 | 4 | 6 | 0.91 | 0.16 | 0.04 |
| UniRef100_C3UZE7 | Germin_like protein 2 *n* = 1 Tax = *Triticum aestivum* RepID = C3UZE7_WHEAT | 2 | 2 | 4 | 0.91 | 0.24 | 0.04 |
| UniRef100_F8S6U4 | Pathogenesis_related protein 1_14 *n* = 1 Tax = *Triticum aestivum* RepID = F8S6U4_WHEAT | 3 | 4 | 24 | 0.91 | 0.12 | 0.00 |
| RFL_Contig6069 | _pep__2:41_1012 | 4 | 5 | 31 | 0.91 | 0.07 | 0.00 |
| RFL_Contig2656 | _pep__2:95_1369 | 5 | 5 | 15 | 0.91 | 0.10 | 0.02 |
| UniRef100_Q7XY23 | Cyc07 *n* = 1 Tax = *Triticum aestivum* RepID = Q7XY23_WHEAT | 5 | 7 | 35 | 0.91 | 0.05 | 0.00 |
| UniRef100_F4Y593 | Heat shock protein 90 *n* = 1 Tax = *Triticum aestivum* RepID = F4Y593_WHEAT | 10 | 12 | 71 | 0.92 | 0.04 | 0.00 |
| UniRef100_Q9ZR33 | Glycosyltransferase 75 *n* = 1 Tax = *Triticum aestivum* RepID = Q9ZR33_WHEAT | 10 | 16 | 138 | 0.92 | 0.05 | 0.02 |
| RFL_Contig1308 | _pep__3:153_1403 | 4 | 5 | 53 | 0.92 | 0.08 | 0.00 |
| UniRef100_F4Y595 | Heat shock protein 90 *n* = 3 Tax = Triticum RepID = F4Y595_WHEAT | 13 | 21 | 63 | 0.92 | 0.04 | 0.00 |
| RFL_Contig5919 | _pep__2:74_865 | 6 | 9 | 93 | 0.92 | 0.03 | 0.00 |
| UniRef100_P83970 | Plasma membrane ATPase *n* = 1 Tax = *Triticum aestivum* RepID = PMA1_WHEAT | 21 | 27 | 185 | 0.92 | 0.03 | 0.00 |
| RFL_Contig4608 | _pep__2:86_1918 | 10 | 14 | 101 | 0.92 | 0.03 | 0.00 |
| RFL_Contig3501 | _pep__2:206_1009 | 3 | 3 | 26 | 0.92 | 0.06 | 0.01 |
| UniRef100_Q1XIR9 | Beta_glucosidase *n* = 1 Tax = *Triticum aestivum* RepID = Q1XIR9_WHEAT | 8 | 9 | 73 | 0.92 | 0.09 | 0.00 |
| UniRef100_Q8GTB7 | Glutathione transferase F1 *n* = 1 Tax = *Triticum aestivum* RepID = Q8GTB7_WHEAT | 3 | 4 | 10 | 0.92 | 0.10 | 0.02 |
| UniRef100_Q8S4X5 | Aquaporin PIP1 *n* = 1 Tax = *Triticum aestivum* RepID = Q8S4X5_WHEAT | 2 | 3 | 21 | 0.92 | 0.18 | 0.01 |
| UniRef100_Q5I7L0 | 60S ribosomal protein L18 *n* = 1 Tax = *Triticum aestivum* RepID = Q5I7L0_WHEAT | 4 | 5 | 23 | 0.92 | 0.07 | 0.01 |
| RFL_Contig3135 | _pep__3:48_797 | 5 | 6 | 25 | 0.93 | 0.04 | 0.00 |
| UniRef100_Q41583 | Initiation factor (Iso)4f p82 subunit *n* = 1 Tax = *Triticum aestivum* RepID = Q41583_WHEAT | 12 | 14 | 60 | 0.93 | 0.05 | 0.00 |
| UniRef100_Q1W681 | Vacuolar proton_ATPase subunit A *n* = 1 Tax = *Triticum aestivum* RepID = Q1W681_WHEAT | 17 | 21 | 126 | 0.93 | 0.04 | 0.00 |
| RFL_Contig2911 | _pep__2:110_1441 | 8 | 8 | 48 | 0.93 | 0.04 | 0.00 |
| UniRef100_C9EF64 | Dehydroascorbate reductase *n* = 1 Tax = *Triticum aestivum* RepID = C9EF64_WHEAT | 8 | 9 | 61 | 0.93 | 0.03 | 0.00 |
| RFL_Contig3954 | _pep__3:162_1994 | 11 | 14 | 111 | 0.93 | 0.03 | 0.00 |

**Table S3.** *Cont.*

| PROTEIN ID | DESCRIPTION | Matched Peptides | FRAMES | HITS | Ratio | SD | *p*-Value |
|---|---|---|---|---|---|---|---|
| UniRef100_Q41629 | ADP_ATP carrier protein 1_ mitochondrial *n* = 1 Tax = *Triticum aestivum* RepID = ADT1_WHEAT | 8 | 12 | 105 | 0.93 | 0.06 | 0.02 |
| UniRef100_Q9SP56 | Glutathione S_transferase *n* = 1 Tax = *Triticum aestivum* RepID = Q9SP56_WHEAT | 4 | 6 | 63 | 0.93 | 0.08 | 0.04 |
| gi_257659932_emb_CBD33996.1_ | unnamed protein product (*Triticum aestivum*) | 4 | 6 | 63 | 0.93 | 0.08 | 0.04 |
| RFL_Contig3640 | _pep__1:91_3054 | 7 | 8 | 40 | 0.93 | 0.08 | 0.00 |
| RFL_Contig5920 | _pep__1:85_912 | 8 | 11 | 52 | 0.93 | 0.05 | 0.04 |
| gi_219914284_emb_CAW74911.1_ | unnamed protein product (*Triticum aestivum*) | 8 | 11 | 65 | 0.93 | 0.05 | 0.00 |
| UniRef100_G1FFN0 | Glutamine synthetase *n* = 2 Tax = Triticeae RepID = G1FFN0_9POAL | 6 | 9 | 40 | 0.93 | 0.11 | 0.01 |
| UniRef100_A9LIN4 | Malic enzyme *n* = 1 Tax = *Triticum aestivum* RepID = A9LIN4_WHEAT | 9 | 10 | 68 | 0.93 | 0.06 | 0.01 |
| UniRef100_C6ETA8 | Class III peroxidase *n* = 1 Tax = *Triticum aestivum* RepID = C6ETA8_WHEAT | 3 | 4 | 10 | 0.93 | 0.05 | 0.00 |
| RFL_Contig4067 | _pep__2:110_1627 | 8 | 9 | 81 | 0.93 | 0.06 | 0.00 |
| RFL_Contig3580 | _pep__3:9_563 | 2 | 3 | 34 | 0.94 | 0.08 | 0.00 |
| UniRef100_C6ES53 | Class III peroxidase *n* = 1 Tax = *Triticum aestivum* RepID = C6ES53_WHEAT | 4 | 6 | 18 | 0.94 | 0.05 | 0.00 |
| UniRef100_Q7XYD5 | Acidic ribosomal protein (Fragment) *n* = 1 Tax = *Triticum aestivum* RepID = Q7XYD5_WHEAT | 4 | 6 | 71 | 0.94 | 0.08 | 0.00 |
| RFL_Contig4097 | _pep__3:123_1553 | 6 | 9 | 22 | 0.94 | 0.07 | 0.05 |
| RFL_Contig3158 | _pep__2:62_631 | 7 | 8 | 79 | 0.94 | 0.05 | 0.00 |
| RFL_Contig3111 | _pep__2:80_682 | 4 | 5 | 51 | 0.94 | 0.11 | 0.00 |
| gi_315113252_pdb_3IZR_F | Chain F_ Localization Of The Large Subunit Ribosomal Proteins Into A 5.5 A Cryo_Em Map of *Triticum aestivum* Translating 80s Ribosome | 3 | 3 | 8 | 0.94 | 0.05 | 0.00 |
| RFL_Contig4002 | _pep__3:30_1007 | 2 | 3 | 13 | 0.94 | 0.12 | 0.01 |
| UniRef100_B9A8E3 | Protein disulfide isomerase *n* = 1 Tax = *Triticum aestivum* RepID = B9A8E3_WHEAT | 13 | 17 | 135 | 0.95 | 0.04 | 0.01 |
| RFL_Contig3538 | _pep__1:112_1083 | 10 | 10 | 76 | 0.95 | 0.03 | 0.00 |
| RFL_Contig3549 | _pep__1:88_426 | 4 | 4 | 56 | 0.95 | 0.09 | 0.00 |
| UniRef100_E3KLJ3 | Calmodulin *n* = 2 Tax = Eukaryota RepID = E3KLJ3_PUCGT | 2 | 2 | 3 | 0.95 | 0.22 | 0.00 |
| gi_296525616_emb_CBM37260.1_ | unnamed protein product (*Triticum aestivum*) | 6 | 7 | 35 | 0.95 | 0.07 | 0.00 |
| UniRef100_Q41534 | ATP synthase subunit beta *n* = 1 Tax = *Triticum aestivum* RepID = Q41534_WHEAT | 19 | 42 | 284 | 0.95 | 0.03 | 0.01 |
| UniRef100_H9ZWY2 | Plastid 3_phosphoglycerate kinase (Fragment) *n* = 1 Tax = *Triticum aestivum* RepID = H9ZWY2_WHEAT | 5 | 5 | 41 | 0.95 | 0.07 | 0.00 |
| RFL_Contig3766 | _pep__1:112_2550 | 12 | 15 | 86 | 0.95 | 0.04 | 0.03 |

**Table S3.** *Cont.*

| PROTEIN ID | DESCRIPTION | Matched Peptides | FRAMES | HITS | Ratio | SD | *p*-Value |
|---|---|---|---|---|---|---|---|
| RFL_Contig3291 | _pep__3:81_1841 | 14 | 16 | 91 | 0.95 | 0.05 | 0.02 |
| RFL_Contig3722 | _pep__3:144_1124 | 2 | 2 | 21 | 0.95 | 0.06 | 0.00 |
| UniRef100_Q1XH05 | Beta_glucosidase *n* = 1 Tax = *Triticum aestivum* RepID = Q1XH05_WHEAT | 9 | 9 | 66 | 0.95 | 0.08 | 0.00 |
| RFL_Contig3927 | _pep__3:123_1715 | 7 | 7 | 38 | 0.95 | 0.05 | 0.01 |
| RFL_Contig2067 | _pep__1:118_1539 | 5 | 5 | 25 | 0.95 | 0.14 | 0.02 |
| RFL_Contig4151 | _pep__3:126_5297 | 32 | 34 | 203 | 0.96 | 0.03 | 0.00 |
| RFL_Contig3262 | _pep__3:150_1172 | 2 | 4 | 22 | 0.96 | 0.18 | 0.03 |
| RFL_Contig308 | _pep__1:73_1512 | 14 | 23 | 146 | 0.96 | 0.05 | 0.00 |
| UniRef100_B2BA41 | Vacuolar proton_ATPase C subunit *n* = 1 Tax = *Triticum aestivum* RepID = B2BA41_WHEAT | 5 | 8 | 65 | 0.96 | 0.07 | 0.00 |
| UniRef100_F8S6V1 | Pathogenesis_related protein 1_21 *n* = 1 Tax = *Triticum aestivum* RepID = F8S6V1_WHEAT | 3 | 4 | 26 | 0.96 | 0.16 | 0.00 |
| gi_257672153_emb_CBD32168.1_ | unnamed protein product (*Triticum aestivum*) | 3 | 11 | 95 | 0.96 | 0.06 | 0.04 |
| gi_257672155_emb_CBD32169.1_ | unnamed protein product (*Triticum aestivum*) | 3 | 11 | 117 | 0.96 | 0.06 | 0.04 |
| UniRef100_Q36813 | NADH dehydrogenase *n* = 1 Tax = *Triticum aestivum* RepID = Q36813_WHEAT | 4 | 4 | 25 | 0.96 | 0.07 | 0.04 |
| UniRef100_Q1PBI3 | Glucose_6_phosphate isomerase *n* = 1 Tax = *Triticum aestivum* RepID = Q1PBI3_WHEAT | 4 | 4 | 34 | 0.96 | 0.02 | 0.00 |
| UniRef100_Q9FS79 | Triosephosphate isomerase *n* = 1 Tax = *Triticum aestivum* RepID = Q9FS79_WHEAT | 9 | 14 | 100 | 0.96 | 0.07 | 0.02 |
| UniRef100_Q53UC8 | Delta1_pyrroline_5_carboxylate synthetase *n* = 1 Tax = *Triticum aestivum* RepID = Q53UC8_WHEAT | 5 | 5 | 29 | 0.97 | 0.08 | 0.05 |
| UniRef100_D5MTD9 | Benzoxazinone:UDP_Glc glucosyltransferase *n* = 1 Tax = *Triticum aestivum* RepID = D5MTD9_WHEAT | 5 | 5 | 14 | 0.97 | 0.11 | 0.02 |
| RFL_Contig3604 | _pep__2:149_973 | 3 | 3 | 28 | 0.97 | 0.09 | 0.00 |
| UniRef100_P55313 | Catalase *n* = 1 Tax = *Triticum aestivum* RepID = CATA2_WHEAT | 9 | 9 | 47 | 0.97 | 0.06 | 0.04 |
| RFL_Contig6066 | _pep__3:69_2492 | 15 | 15 | 111 | 0.98 | 0.04 | 0.00 |
| UniRef100_D5MTF8 | Beta_glucosidase *n* = 1 Tax = *Triticum aestivum* RepID = D5MTF8_WHEAT | 11 | 12 | 89 | 0.98 | 0.08 | 0.00 |
| RFL_Contig3091 | _pep__3:108_1586 | 11 | 11 | 57 | 0.98 | 0.06 | 0.05 |
| RFL_Contig1587 | _pep__2:131_1375 | 13 | 15 | 83 | 0.99 | 0.04 | 0.00 |
| UniRef100_H9ZWY1 | Plastid 3_phosphoglycerate kinase (Fragment) *n* = 1 Tax = *Triticum aestivum* RepID = H9ZWY1_WHEAT | 4 | 4 | 10 | 0.99 | 0.09 | 0.00 |
| RFL_Contig4165 | _pep__1:100_963 | 4 | 4 | 22 | 0.99 | 0.12 | 0.05 |

## References

1. Murashige, T.; Skoog, F. A revised medium for rapid growth and bioassays with tobacco tissue cultures. *Physiol. Plant.* **1962**, *15*, 473–497.

© 2014 by the authors; licensee MDPI, Basel, Switzerland. This article is an open access article distributed under the terms and conditions of the Creative Commons Attribution license (http://creativecommons.org/licenses/by/4.0/).
